# MNKs act as a regulatory switch for eIF4E1 and eIF4E3 driven mRNA translation in DLBCL

**DOI:** 10.1038/ncomms6413

**Published:** 2014-11-18

**Authors:** Ari L. Landon, Parameswary A. Muniandy, Amol C. Shetty, Elin Lehrmann, Laurent Volpon, Simone Houng, Yongqing Zhang, Bojie Dai, Raymond Peroutka, Krystyna Mazan-Mamczarz, James Steinhardt, Anup Mahurkar, Kevin G. Becker, Katherine L. Borden, Ronald B. Gartenhaus

**Affiliations:** 1 Marlene & Stewart Greenebaum Cancer Center, Department of Medicine, University of Maryland, Baltimore, Maryland 21201, USA; 2 Veterans Administration Medical Center, Baltimore, Maryland 21201, USA; 3 Institute for Genome Sciences, University of Maryland School of Medicine, Baltimore, Maryland 21201, USA; 4 Gene Expression and Genomics Unit, National Institute on Aging, National Institutes of Health, Baltimore, Maryland 21224, USA; 5 Institute for Research in Immunology and Cancer, Department of Pathology and Cell Biology, Université de Montréal, Montréal, Québec, Canada H3T 1J4

## Abstract

The phosphorylation of eIF4E1 at serine 209 by MNK1 or MNK2 has been shown to initiate oncogenic mRNA translation, a process that favours cancer development and maintenance. Here, we interrogate the MNK-eIF4E axis in diffuse large B-cell lymphoma (DLBCL) and show a distinct distribution of MNK1 and MNK2 in germinal centre B-cell (GCB) and activated B-cell (ABC) DLBCL. Despite displaying a differential distribution in GCB and ABC, both MNKs functionally complement each other to sustain cell survival. MNK inhibition ablates eIF4E1 phosphorylation and concurrently enhances eIF4E3 expression. Loss of MNK protein itself downregulates total eIF4E1 protein level by reducing eIF4E1 mRNA polysomal loading without affecting total mRNA level or stability. Enhanced eIF4E3 expression marginally suppresses eIF4E1-driven translation but exhibits a unique translatome that unveils a novel role for eIF4E3 in translation initiation. We propose that MNKs can modulate oncogenic translation by regulating eIF4E1-eIF4E3 levels and activity in DLBCL.

## Abstract (web summary)

Diffuse large B-cell lymphoma (DLBCL) is a highly aggressive and heterogeneous type of non-Hodgkin’s lymphoma. Here the authors demonstrate that the differential regulation of eIF4E1 and eIF4E3 by the MAPK-interacting kinases is involved in DLBCL aetiology through modification of the cellular translatome.

## Graphical abstract

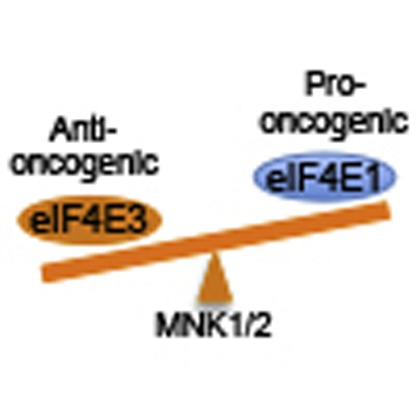

There are multiple aetiologies to cancer development and maintenance, which essentially exert a selection pressure for enhancing oncogenic gene expression and/or reducing tumour suppressor activity. The transformation process leading up to malignancy is a result of a biased cellular homeostasis that favours increased and uncontrolled growth and proliferation. One of the least explored yet fundamentally important cellular processes that controls oncogenic transcript selection and expression is mRNA translation. Dysregulation of the translation process can alter the cellular landscape that can lead to cancer initiation, maintenance, progression, invasion and metastasis^1^,^2^. Cap-dependent translation is the primary mechanism of mRNA translation in eukaryotic cells. The most common member of the cap-dependent translation machinery that is often upregulated in cancer is eukaryotic translation initiation factor 4E 1 (eIF4E1), a 25-kDa protein that serves to initiate cap-dependent translation via mRNA cap binding, a highly regulated rate-limiting step in translation initiation. eIF4E1 functions to bridge mRNA to the ribosome via the eIF4F complex assembly^1^,^3^,^4^,^5^,^6^,^7^. The oncogenic potential of eIF4E1 has been characterized in many model systems and is emerging as an attractive therapeutic target for cancer, giving rise to eIF4E/eIF4E-cap inhibitors like ISIS183750 and ribavirin in clinical trials^8^.

The sole upstream regulators of eIF4E1 phosphoactivation are mitogen-activated protein kinase (MAPK) interacting kinases 1 and 2 (MNK1 and MNK2), which operate by phosphorylating eIF4E1 at serine 209 (S209) when both eIF4E1 and MNK are positioned in close proximity to each other on binding to the scaffolding protein, eIF4G^9^. The regulation of MNKs, in turn, is modulated via ERK and p38 MAPKs, as predicted by the presence of a MAPK-binding domain in the C terminus of the longer alternate splice form of both MNKs^10^,^11^,^12^. Although most work has proposed that ERK and p38 MAPKs function upstream of MNKs, a very recent work by Maimon *et al.*^13^ proposed a downstream activation of p38 MAPKs by the longer isoform of MNK2 (that is, MNK2a). These authors described a tumour suppressive role for this MNK2a-p38 MAPKs activity and a pro-oncogenic role for p38-MNK2b axis^13^.

MNK2 exhibits higher basal activity, poor upstream control by ERK or p38 MAPKs, and is emerging as a strong chemoresistant candidate to rapalogues and gemcitabine treatments^12^,^14^,^15^,^16^,^17^. A number of studies have established the importance of the MNK-eIF4E1 axis in several human malignancies. Surprisingly, mice lacking both MNKs exhibited normal survival with no obvious phenotype^18^. Although this finding highlighted the dispensable nature of MNK activity under normal physiological conditions, other studies employing both *in vitro* and *in vivo* approaches have provided substantial evidence that downregulation of MNK or eIF4E1 phosphorylation in cancer is favourable for tumour regression^15^,^19^,^20^.

There are three members in the eIF4E family, where two members, eIF4E1 and eIF4E2, have been shown to bind the 7-methyl-guanosine (m^7^G)-cap using the classical aromatic sandwich model. Owing to the weak cap-binding ability and lack of significant eIF4G association reported in earlier studies, eIF4E2 was not believed to initiate translation in normal cells^7^,^21^. However, recent studies have demonstrated eIF4E2-directed translation under low-oxygen conditions, giving rise to a new perspective for eIF4E2-modulated protein synthesis in tumour hypoxia^7^,^21^,^22^,^23^. The third member of this family, eIF4E3, was not believed to have cap-binding ability as its primary structure lacks one of the two aromatic residues needed to bind m^7^G-cap. However, a recent intriguing finding by Osborne *et al.*^7^ using biophysical approaches showed that eIF4E3 is indeed able to bind m^7^G-cap in an atypical manner and exerts tumour suppressive effects in cells.

Here, we comprehensively interrogate the MNK-eIF4E axis in DLBCL, a highly aggressive and heterogeneous non-Hodgkin’s lymphoma. We show that both MNKs complement each other for cell survival despite exhibiting a differential distribution in DLBCL subtypes. MNKs, via its kinase-dependent or independent roles, alter the ability for eIF4E1 and eIF4E3 to bind the mRNA cap structure, thus, displaying a capacity to ‘switch’ the cellular translatome.

## Results

### MNK1 and MNK2 are differentially expressed in DLBCL subtypes

**Figure 1:**
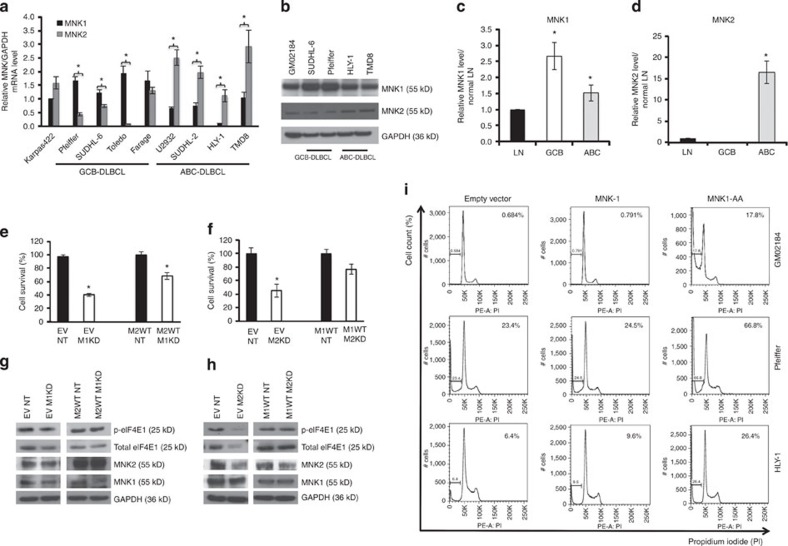
MNK1 and MNK2 expression in non-malignant (GM02184), malignant (DLBCL) cell lines and primary tissue samples. (**a**) RT–qPCR analysis of MNK1 (black bar) and MNK2 (grey bar) total mRNA in ABC- and GCB-DLBCL cell lines compared with GAPDH mRNA (mean±s.e.m., *n*=3, *P-value of Student’s *t*-test <0.05). (**b**) Western blot analysis of MNK1 and MNK2 protein expression in ABC- and GCB-DLBCL cell lines with GAPDH loading control. Densitometry analysis is shown in Supplementary Fig. 1a. (**c**,**d**) RT–qPCR analysis of (**c**) MNK1 or (**d**) MNK2 mRNA levels in total RNA extracted from primary tissue microarray preclassified as normal lymph node (LN, black bar), GCB- (white bar) or ABC- (grey bar) DLBCL, relative to (**c**) MNK1 or (**d**) MNK2 levels in normal lymph node (mean±s.e.m., *n*=3, *P-value of Student’s *t*-test <0.05). (**e**) Survival-rescue experiment of HLY-1 DLBCL, 48 h after transduction of non-target (NT, black bar) or MNK1 shRNA (M1KD, white bar) in both empty vector (EV) and MNK2 WT (M2WT) overexpressing cells, measured by trypan blue exclusion assay (mean±s.d., *n*=3, *P-value of Student’s *t*-test <0.05). (**f**) Survival-rescue experiment of HLY-1 DLBCL, 48 h after transduction of non-target (NT, black bar) or MNK2 shRNA (M2KD, white bar), in both empty vector (EV) and MNK1 WT (M1WT) overexpressing cells, measured by trypan blue exclusion assay (mean±s.d., *n*=3, *P-value of Student’s *t*-test <0.05). (**g**) Western blot analysis of total and phospho-eIF4E1(S209) as well as MNK1, MNK2 and GAPDH for MNK1 KD rescue experiment from Fig. 1e in HLY-1 expression mutants. Densitometry analysis is shown in Supplementary Fig. 1b. (**h**) Western blot analysis of total and phospho-eIF4E1(S209) as well as MNK1, MNK2 and GAPDH for MNK2 KD rescue experiments from Fig. 1f in HLY-1 expression mutants. Densitometry analysis is shown in Supplementary Fig. 1c (**i**) Representative cell cycle analysis from three independent experiments of stable cell lines expressing empty vector, wild-type MNK1 (MNK1) or MNK1 phosphonull mutant (MNK1-AA) in non-malignant B-cell line GM02184 and DLBCL cell lines HLY-1 and Pfeiffer. Full immunoblots are shown in Supplementary Fig. 10.

The status of MNKs, their distribution and activity in DLBCL have not been described to date. Data mining from previously reported gene expression analyses demonstrate that DLBCL samples can exhibit a varied range of MNK1 and MNK2 expressions. However, there are no comparisons made to evaluate the relative abundance of MNK1 or MNK2 in any individual sample in a given study population (Supplementary Table 1, www.oncomine.org). We analysed a panel of DLBCL cell lines that are characterized as either ABC or GCB origin for MNK1 and MNK2 expression levels via semi-quantitative real-time PCR (RT-qPCR)^24^. MNK1 was expressed in both ABC- and GCB-DLBCL; however, a significantly stronger expression of MNK1 was evident in all GCB-DLBCL cell lines tested (Fig. 1a). In contrast, ABC-DLBCL exhibited a stronger MNK2 expression. Western blot analysis of these cell lines further confirmed that the discrete MNK distribution pattern was also consistent at the protein level (Fig. 1b). We extracted RNA from paraffin-embedded primary lymphoma samples for RT-qPCR analysis^25^. These lymphoma samples were previously scored as ABC- or GCB-DLBCL by an independent pathologist based on immunohistochemical characterization^26^. Primary patient samples of GCB-DLBCL exhibited enhanced MNK1 expression consistent with cell line data, and, notably, undetectable MNK2 levels (Fig. 1c,d). MNK2 was only detectable at measureable levels in primary ABC-DLBCL samples, in addition to MNK1 (Fig. 1d). Given the distinct MNK1 and MNK2 distribution pattern in ABC and GCB, we next asked whether both MNKs were important for cellular viability and one could complement the other in promoting survival. We found that reducing the expression of either MNK1 or MNK2 significantly reduced overall cell survival as reported previously^27^ and, in the same experiments, when one MNK member is overexpressed while the other is knocked down (that is, when MNK2 is overexpressed and MNK1 is knocked down simultaneously, or vice versa), cell survival as well as phosphorylation of eIF4E1 in either case were significantly rescued (Fig. 1e–h). The differential expression in ABC- and GCB-DLBCL notwithstanding, we find that MNK1 and MNK2 can complement each other functionally to maintain cellular survival. Similarly, enforced expression of MNK1-AA mutant (MNK1-phosphonull) that cannot be phosphorylated for downstream activity caused significant cell death in GM02184, a non-malignant B-cell line, and to a much higher degree in DLBCL cell lines Pfeiffer (GCB-DLBCL) and HLY-1 (ABC-DLBCL) (Fig. 1i), suggesting that expression of an inactive MNK (MNK1-AA) acts as a dominant negative mutant in both MNK1- or MNK2-expressing cells, reaffirming a common or competitive role for both MNKs. Immunohistochemistry staining of a primary human lymphoma tissue microarray revealed significant elevation of p-eIF4E1 staining in DLBCL in comparison with normal tissues, regardless of the ABC or GCB classifications (normal LN=20, ABC=35 and GCB=11 samples) (Supplementary Fig. 1d).

### MNKs are regulated by p38 and not ERK in DLBCL

**Figure 2:**
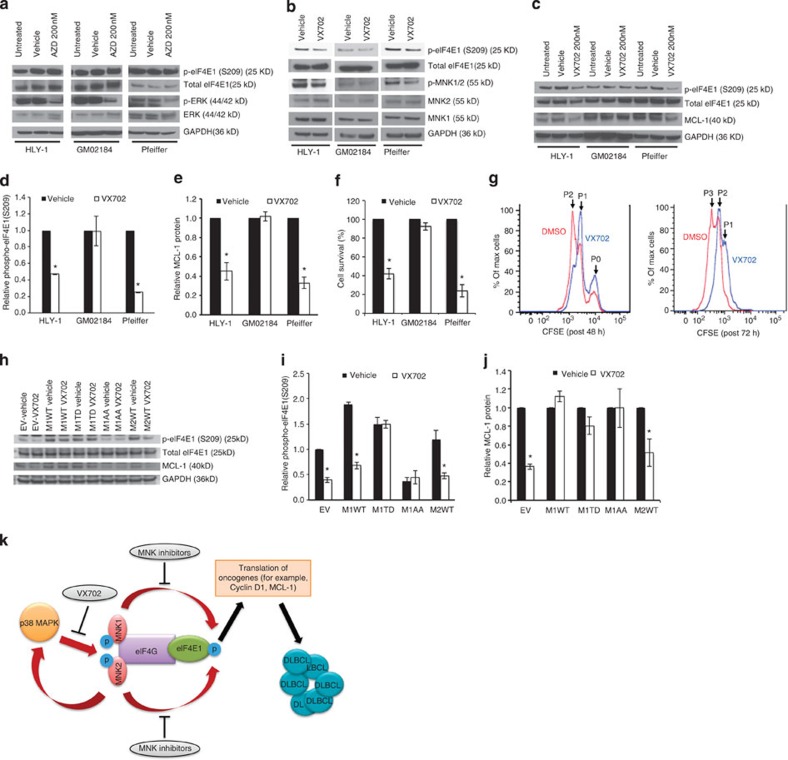
p38 is the primary regulator of MNK-driven eIF4E1 phosphorylation in DLBCL. (**a**) Western blot analysis of total and phospho-eIF4E1(S209) as well as total and phospho-ERK after 24 h treatment with an MEK inhibitor AZD6244 (AZD 200 nM), compared with untreated and vehicle controls in HLY-1, GM02184 and Pfeiffer cell lines. Densitometry analysis is shown in Supplementary Fig. 1e. (**b**) Western blot analysis of phospho-MNKs (antibody detects p-MNK1 and p-MNK2), total MNK1, total MNK2, total and phospho-eIF4E1(S209) after 1 h treatment with a p38 inhibitor VX702 (200 nM). Densitometry analysis is shown in Supplementary Fig. 1f. (**c**) Western blot analysis of total and phospho-eIF4E1 and MCL-1 post 4 h treatment with a p38 inhibitor VX702. (**d**) Densitometry analysis of phospho-eIF4E1(S209) and (**e**) MCL-1 band intensity of western blot in Fig. 2c, relative to GAPDH following 4 h treatment with vehicle (black bar) or 200 nM VX702 (white bar). Mean±s.d., *n*=3, *P-value of Student’s *t*-test <0.05. (**f**) Trypan blue exclusion assay of HLY-1, GM02184 and Pfeiffer cells after 72 h treatment with vehicle (black bar) or 200 nM VX702 (white bar). Mean±s.d., *n*=3, *P-value of Student’s *t*-test <0.001. (**g**) Representative CFSE analysis of HLY-1 cells 48 and 72 h post treatment with 200 nM VX702 (blue line) or vehicle (DMSO; red line). P0 denotes initial population, while P1, P2 and P3 denote subsequent daughter cell populations. Three independent experiments are shown in Supplementary Fig. 4a. (**h**) Western blot showing total and phospho-eIF4E1(S209) as well as MCL-1 with or without 4 h treatment with 200 nM VX702 in HLY-1 cells expressing empty vector (EV), MNK1-wildtype (M1WT), MNK1-phosphomimetic (M1TD), MNK1-phosphonull (M1 AA), and MNK2-wildtype (M2WT). (**i**,**j**) Densitometry analysis showing relative band intensity of (**i**) phospho-eIF4E1(S209) or (**j**) MCL-1 to GAPDH in HLY-1 cells treated with vehicle (black bar) or 200 nM VX702 (white bar) for 4 h of immunoblot in Fig. 2h (mean±s.d., *n*=3, *P-value of Student’s *t*-test <0.05). (**k**) Summarizing figure illustrating the p38-regulated MNK-dependent eIF4E1 phosphorylation in DLBCL cells. Cartoon depicts two potential modes of disrupting the p38-MNK-eIF4E1 axis in DLBCL, that are, via the use of p38 or MNK inhibitors. Full immunoblots are shown in Supplementary Fig. 10.

Two isoforms of each MNK have been identified in human; the longer isoforms, termed MNK1a and MNK2a, contain the MAPK-binding site in the C terminus, which allows binding and activation of MNKs by either ERK or p38 MAPKs. Although MNK1 and MNK2 show differences at the C terminus, both MNKs share high homology in the central catalytic domain and contain phosphorylation sites for the activation of its kinase function. Given that MNK activation is associated with either ERK or p38-MAPK as upstream regulators, we probed these two targets as potential regulators of MNK activity in an array of DLBCL cell lines and in GM02184. We find that MEK inhibition (using AZD6244), which reduced ERK phosphorylation, had no impact on MNK kinase activity in DLBCL, evidenced by no change in eIF4E1-S209 phosphorylation at any time point tested, ranging from 45 min to 72 h (Fig. 2a and Supplementary Figs 1a,2). We concurrently tested the ERK2 inhibitor, no.76 (3-(2-aminoethyl)-5-((4-ethoxyphenyl) methylene)-2,4-thiazolidine-dione, HCl) and found corroborative results showing no impact of ERK on MNK activity in DLBCL (Supplementary Fig. 2). Others have demonstrated protein phosphatase 2 (PP2A) to dephosphorylate MNK and eIF4E1 (ref. 28). We explored PP2A activation using FTY720 as a potential MNK inhibitor and found no impact on eIF4E1-S209 phosphorylation (Supplementary Fig. 3).

We next examined the impact of p38 inhibition on MNK activity. Inhibition of p38 using VX702 showed a significant reduction of MNK and eIF4E1 phosphorylation within 1 h post treatment (Fig. 2b and Supplementary Fig. 1b) and sustained eIF4E1 phosphorylation reduction 4 h after treatment (Fig. 2c,d). p38 inhibition also resulted in reduced MCL-1, further confirming the effective disruption of eIF4E1-driven translation via manipulation of the p38-MNK-eIF4E1 axis (Fig. 2c,e). GM02184 showed a moderate but significant p-eIF4E1 reduction at 1 h treatment (Fig. 2b and S1b), but rapidly regained eIF4E1 phosphorylation by 4 h with no significant changes in MCL-1. p38 inhibitor treatment of DLBCL cells resulted in growth inhibition, showing reduced viable cell number by trypan exclusion assay, without significant cell death (Fig. 2f). We performed a CFSE-based assay to evaluate cell proliferative response. As illustrated in Fig. 2g (and Supplementary Fig. 4a), p38 inhibition resulted in a slower rate of cell proliferation, trailing at least one cell cycle behind vehicle-treated HLY-1 cells after 72 h. We concurrently performed cell cycle analysis of cells treated with vehicle or VX702 and found a marginal accumulation in the S-phase (Supplementary Fig. 4b), however, with a significantly reduced BrdU incorporation (Supplementary Fig. 4c), demonstrating a reduced S-phase efficiency and prolonged cell cycle length following p38 inhibition in DLBCL.

To affirm the direct effect of p38 on the MNK-eIF4E1 axis, we generated cell lines stably expressing wild-type MNK1 and MNK2, as well as MNK1-TD (MNK1 phosphomimetic) and MNK1-AA (MNK1-phosphonull) mutants in HLY-1 parent line. Inhibition of p38 exerted significant effects on eIF4E1-S209 phosphorylation in cell lines expressing wild-type MNK1 or MNK2, consistent with previous reports^29^. However, in the MNK1-TD and MNK1-AA mutants, where MNK phosphorylation cannot be manipulated by p38, eIF4E1-S209 phosphorylation was not affected by p38 inhibitor, further confirming that p38 activity on MNKs and subsequent eIF4E1-S209 phosphorylation is dependent on its ability to phosphorylate MNK, and that p38 activity is upstream to MNKs in DLBCL (Fig. 2h,i). In these experiments, we also probed for MCL-1 as a readout for eIF4E1 activity as previously reported^19^. We found that MCL-1 was reduced in control and MNK2 overexpression HLY-1 cells treated with VX702. However, MNK-TD and MNK-AA mutants that have constitutively activated or inactivated phosphorylation site that bypass regulation of p38 did not exhibit a reduction in MCL-1, consistent with unchanged eIF4E1 phosphorylation level (Fig. 2i,j). Both MNK1 and MNK2 cells exhibited a significant reduction in p-eIF4E1 levels; however, only MNK2 cells showed a robust reduction in MCL-1. Both MNK1 and MNK2 cells exhibit higher basal levels of MNKs and p-eIF4E1; thus, one can argue that the effects of p38 inhibition were not sufficient for efficient reduction in MCL-1 readout. Although the fact that MNK2 cells showed significant MCL-1 reduction may, at first, appear to counter the argument, this reduction could be an effect of recently reported MNK2 upstream regulation of p38 (ref. 13). p38 inhibition using VX702 causes MNK2 inhibition, which in turn exerts a looped positive feedback of p38 inhibition to further accentuate the cycle of inhibition (Fig. 2k).

The mechanistic basis for the preferential upstream regulation of MNKs by p38 MAPKs and not ERK in DLBCL is still unknown. The regulation of MNKs could be tissue specific and be subjected to co-expression of other factors in the cell. Consistently, we find our data to be in agreement with murine B cells that are also regulated primarily by p38 MAPKs^30^.

### MNKs differentially regulate eIF4E1 and eIF4E3

**Figure 3:**
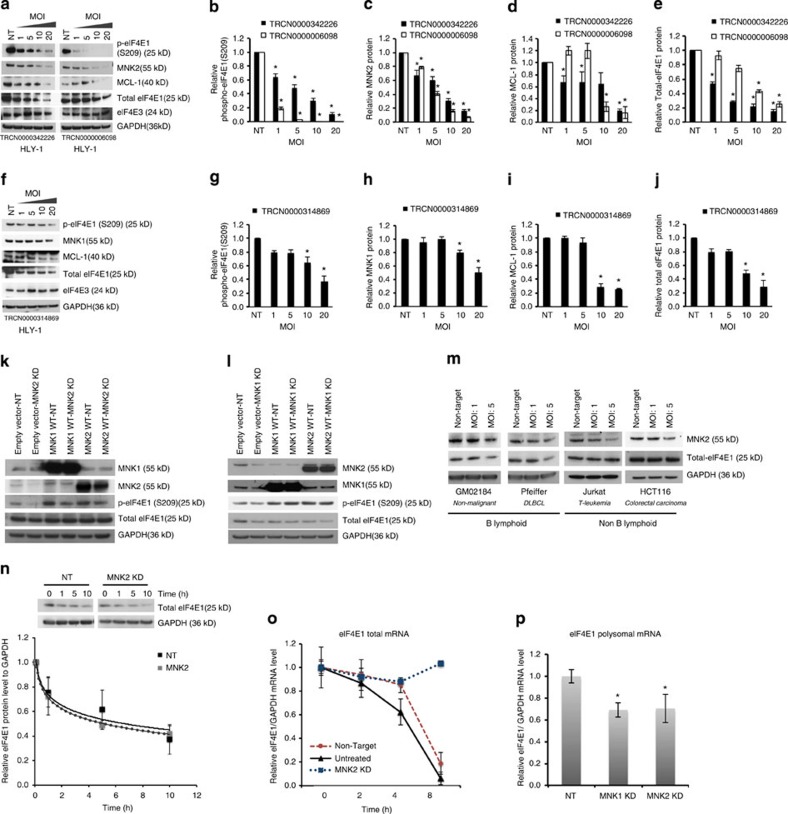
MNK expression regulates eIF4E1 in a dose-dependent manner. (**a**) Western blot showing total and phospho-eIF4E1(S209), MCL-1, eIF4E3 and MNK2 of HLY-1 cells 48 h post-transduction with two pre-validated MNK2 shRNAs at MOI of 1 to 20. (**b**–**e**) Densitometry analysis showing band intensity relative to GAPDH of (**b**) phospho-eIF4E1(S209), (**c**) MNK2, (**d**) MCL-1 and (**e**) total eIF4E1 in pre-validated MNK2 shRNAs (black bar, TRCN0000342226 and white bar, TRCN0000006098) in Fig. 3a. Mean±s.d., *n*=3, *P-value of Student’s *t*-test <0.05. (**f**) Western blot showing total and phospho-eIF4E1(S209), MCL-1, eIF4E3 and MNK1 of HLY-1 cells 48 h post-transduction with a pre-validated MNK1 shRNA at MOI of 1 to 20. (**g**–**j**) Densitometry analysis showing band intensity relative to GAPDH of (**g**) phospho-eIF4E1(S209), (**h**) MNK1, (**i**) MCL-1 and (**j**) total eIF4E1 in MNK1 shRNA transduction as in Fig. 3f. Mean±s.d., *n*=3, *P-value of Student’s *t*-test <0.05. (**k**,**l**) Western blot analysis of HLY-1 stable cell lines expressing MNK1 or MNK2, 48 h after transduction with non-target (NT) shRNA, (**k**) MNK2 shRNA (MNK2 KD) at MOI of 10, or (**l**) MNK1 shRNA (MNK1 KD) at MOI of 20. Densitometry analysis of Fig. 3k,l is shown in Supplementary Fig. 5a,b respectively. (**m**) Western blot showing MNK2 and total eIF4E1 in various cell lines 48 h post-transduction with MNK2 shRNA at MOI of 1 and 5. Densitometry analysis of Fig. 3m is in Supplementary Fig. 5c. (**n**) Top: western blot of HLY-1 cells treated with cycloheximide (100 μg ml^−1^) after MNK2 shRNA transduction at MOI of 10, compared with non-target shRNA (NT) control. Bottom: densitometry analysis of eIF4E1 protein level over time for non-target (NT, black squares) and MNK2 (grey squares) transduced HLY-1 cells. Values are mean±s.d., *n*=3, with logarithmic-fit curve (**o**) RT-qPCR analysis showing total eIF4E1 mRNA level relative to GAPDH expression in HLY-1 cells untreated (black triangles), and transduced with either non-target (red circles) or MNK2 shRNA (MNK2 KD, blue squares) viral particles, followed by actinomycin-D treatment (mean±s.d., *n*=3). (**p**) RT-qPCR analysis measuring eIF4E1 mRNA level in polysomal fractions, 48 h after knockdown by MNK1 or MNK2 shRNA (mean±s.d., *n*=3, *P-value of Student’s *t*-test <0.05). Full immunoblots are shown in Supplementary Fig. 10.

There have previously been a number of conflicting views on the importance of eIF4E1 phosphorylation status in initiating translation under basal conditions^18^,^19^,^20^. To explore how MNK activity and variable protein expression affect eIF4E1 and mRNA translation in DLBCL, we performed the following experiments. First, we established a serial knockdown of MNK1 and MNK2 proteins in the HLY-1 cell line. Unexpectedly, we found that eIF4E1 protein level decreased proportionally to MNK knockdown in a dose-dependent manner. These data were consistent across all three shRNAs designed and validated for MNK1 and MNK2 (Fig. 3a–j). To attest against an off-target shRNA effect, we transduced MNK1 or MNK2 shRNA in HLY-1 cell lines that were overexpressing MNK1 and MNK2 to introduce the same amount of shRNA viral particles in the cells. The amount of shRNA introduced could not sufficiently reduce the abundant MNK levels. We found that the decrease in eIF4E1 levels associated with MNK1/2 knockdowns was completely abolished, further assuring that the effects of the shRNAs are not directly on eIF4E1 and are mediated via protein levels of MNKs (Fig. 3k,l, Supplementary Fig. 5a,b). In addition to confirming MNK-dependent eIF4E1 protein level, these data also strengthen the point that MNK1 and MNK2 could complement each other in maintaining eIF4E1 levels *in vivo* (Figs 1e–h and 3k,l). We compared our findings with the previously reported MNK knockout mouse data^18^. Ueda *et al.*^18^ reported neither a phenotype nor changes in total eIF4E1 level in these mice. However, when these mice were crossed with *PTEN*-deficient T-lymphoma-forming mice (tPTEN), the resultant MNK knockout/tPTEN mice exhibited delayed lymphomagenesis. These data, presented more recently by Ueda *et al.*^20^, revealed a possible explanation for the discrepancy observed with our findings^20^. Basal eIF4E1 level and phosphorylation in the tPTEN/MNK WT (MNK1+/−, MNK2+/−) tumour were significantly higher than the WT thymus samples, possibly showing a higher dependency on the MNK-eIF4E1 pathway in these tumours. However, in the same tumours, when MNK1, MNK2 or both were eliminated, 50% (1/2), 100% (1/1) and 66% (2/3) of tumours, respectively, exhibited reduced total eIF4E1 protein level in comparison with WT MNKs in the tPTEN background. This raised the possibility that eIF4E1 regulation by MNK protein is potentially enhanced and is more prominent when the MNK-eIF4E1 axis becomes crucial for survival. We interrogated a series of transformed cell lines to determine whether this phenomenon was preserved across different tissues. As illustrated in Fig. 3m (and Supplementary Fig. 5c), we find that the MNK-dependent eIF4E1 protein level was consistent in two other B-lymphoid origin cell lines; however, in T-leukaemia (Jurkat) and colorectal carcinoma (HCT116) cells, the reduction in MNK level did not affect the eIF4E1 levels.

To investigate the mechanism of MNK-dependent eIF4E1 expression, we measured eIF4E1 protein half-life with and without MNK knockdown. Cells were first transduced with either MNK2 shRNA or non-target (NT) control viruses. Forty-eight hours post transduction, cells were treated with cycloheximide to halt translation/protein synthesis and then samples were collected at various time points to measure eIF4E1 protein levels. Following immunoblotting and probing for eIF4E1, we performed densitometry on triplicate experiments to calculate protein half-life. We found that eIF4E1 protein half-life was ~4.5 h, as reported previously, and the half-life was not affected by MNK knockdown (Fig. 3n). These data suggest that MNK-dependent eIF4E1 depletion was not related to accelerated protein degradation or reduced stability determined by MNK. However, the reduction of eIF4E1 with MNK knockdown was eliminated after inhibition of mRNA translation with cycloheximide, suggesting that MNK-dependent eIF4E1 depletion was associated with mRNA translation or stability. Total mRNA levels of eIF4E1 in untreated, non-target (NT) control and MNK2 knockdown were subsequently investigated. We treated cells with actinomycin-D to inhibit transcription of new mRNA templates and collected samples at various time points post treatment. We found no significant reduction in eIF4E1 mRNA levels on MNK2 knockdown, strongly arguing against mRNA instability as a reason for the eIF4E1 protein depletion seen with MNK knockdown (Fig. 3o, Supplementary Fig. 5d). In fact, we saw greater mRNA stability with MNK knockdown. Taken together, these data showed that the reduction of eIF4E1 protein expression with MNK knockdown was not mediated via altered protein or mRNA stability. We analysed the heavy polysomal fraction from sucrose density gradient separation containing highly translated polysome-bound mRNA transcripts. Intriguingly, we found that while no significant difference in total mRNA was detected, eIF4E1 mRNA level in the polysomal fraction was significantly reduced (Fig. 3o–p). These experimental findings are consistent with MNKs playing a novel role in eIF4E1 translation and protein expression in addition to its well-recognized kinase activity in phosphorylating eIF4E1.

**Figure 4:**
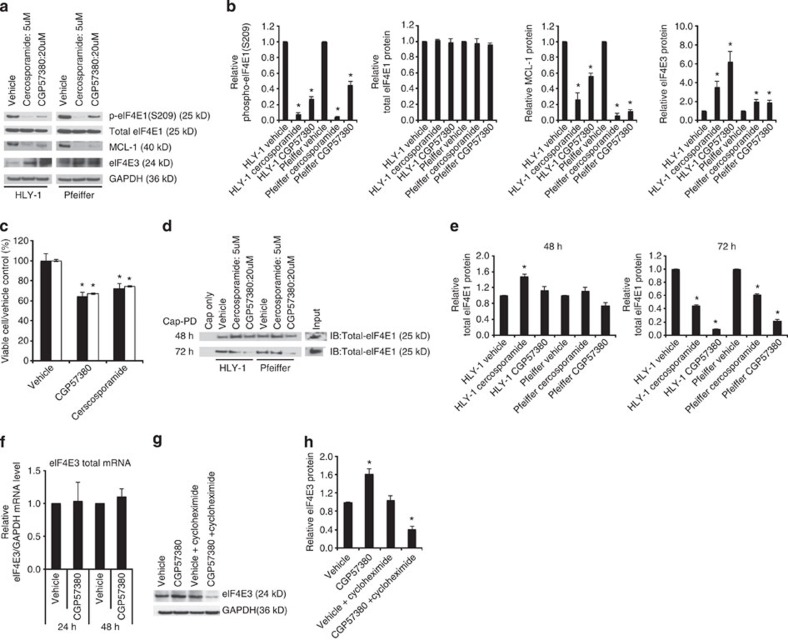
MNK kinase activity inhibition on eIF4E1 and eIF4E3. (**a**) Western blot showing total and phospho-eIF4E1(S209), MCL-1 and eIF4E3 in HLY-1 and Pfeiffer DLBCL cell lines after 48 h treatment with MNK inhibitors, cercosporamide and CGP57380. (**b**) Densitometry analysis showing band intensity relative to GAPDH of phospho-eIF4E1(S209), total eIF4E1, MCL-1 and eIF4E3 proteins in Fig. 4a. Mean±s.e.m., *n*=3, *P-value of Student’s *t*-test <0.05. (**c**) Trypan blue exclusion assay of HLY-1 (black bars) and Pfeiffer (white bars) cells after 48 h treatment with vehicle, 5 μM cercosporamide or 20 μM CGP57380, and values normalized to vehicle. Mean±s.d., *n*=3, *P-value of Student’s *t*-test <0.05. (**d**) Western blot analysis following cap-pull down of HLY-1 and Pfeiffer cells following 48 and 72 h treatment with MNK inhibitors, probed for total eIF4E1. (**e**) Densitometry analysis showing band intensity relative to GAPDH of total eIF4E1 after 48 h (left panel) and 72 h (right panel) treatment with 5 μM cercosporamide or 20 μM CGP57380, as in Fig. 4d. Mean±s.e.m., *n*=3, *P-value of Student’s *t*-test <0.05. (**f**) RT-qPCR analysis measuring total eIF4E3 mRNA level in HLY-1 cells, 24 or 48 h after treatment with CGP57380 (20 μM). Values are mean±s.e.m., *n*=3, *P-value of Student’s *t*-test <0.05). (**g**) Western blot showing eIF4E3 level in HLY-1 cells after 48 h treatment with 20 μM CGP57380 and/or 100 μg ml^−1^ cycloheximide. (**h**) Densitometry analysis showing band intensity relative to GAPDH of eIF4E3 level in HLY-1 cells after 48 h treatment with 20 μM CGP57380 and 100 μg ml^−1^ cycloheximide, alone or in combination, as in Fig. 4f. Mean±s.e.m., *n*=3, *P-value of Student’s *t*-test <0.05. Full immunoblots are shown in Supplementary Fig. 10.

We investigated MNK kinase inhibition in DLBCL using two well-characterized MNK inhibitors: cercosporamide and CGP57380. Cercosporamide has been shown to be a more selective and potent inhibitor of MNKs in comparison with CGP57380. However, both inhibitors also been shown to exhibit a broader effect on several other kinases^10^,^29^,^31^. In accordance with previous reports, both inhibitors exerted significant effect on MNK kinase activity, resulting in reduced eIF4E1-S209 phosphorylation with no changes in eIF4E1 total protein level. Thus, we find that MNK in its physical form has a kinase-independent role in maintaining the cellular eIF4E1 level, while the kinase-dependent function of MNK is important for eIF4E1 phosphorylation. As an eIF4E1-mediated translation readout, we also found MCL-1 was reduced in both treatments. Unexpectedly, we found eIF4E3, another eIF4E family member with significant cap-binding potential, was increased following MNK inhibitor treatment. To further probe eIF4E3 upregulation, we took advantage of the differential readouts of both cercosporamide and CGP57380 on both HLY-1 and Pfeiffer cell lines. Cercosporamide showed a stronger reduction in eIF4E1-S209 phosphorylation, while CGP57380 (ref. 10) showed a stronger increase in eIF4E3 expression (Fig. 4a,b). MNK inhibition using either compound resulted in significant cell death (Fig. 4c). We performed an m^7^G-cap-binding assay in DLBCL cell lines following treatment with both MNK inhibitors. We found that cercosporamide treatment, which showed a stronger reduction in eIF4E1-S209 phosphorylation, did not affect eIF4E1 m^7^G-cap-binding ability, arguing against the need for eIF4E1-S209 phosphorylation for enhanced cap binding. On the contrary, treatment with CGP57380, having shown a substantial increase in eIF4E3 expression, resulted in a significant decrease in eIF4E1 binding to m^7^G-cap, proposing a potential role for eIF4E3 in affecting eIF4E1 cap binding (Fig. 4d,e).

The increase in eIF4E3 following MNK kinase inhibitor was not due to changes in total mRNA level (Fig. 4f). To understand the mechanism of eIF4E3 upregulation, we co-treated cells with an MNK inhibitor, CGP57380, that caused eIF4E3 upregulation, with cycloheximide to halt mRNA translation. Halting translation significantly eliminated eIF4E3 upregulation, strongly suggesting that observed the eIF4E3 upregulation was mediated via an mRNA translation process (Fig. 4g,h). Here, we report that reduction in MNK protein level suppressed eIF4E1 translation, while inhibition of MNK kinase activity significantly reduced eIF4E1-S209 phosphorylation and increased eIF4E3 levels in DLBCL cell lines.

### Unphosphorylated eIF4E1 enhances eIF4E3 expression

**Figure 5:**
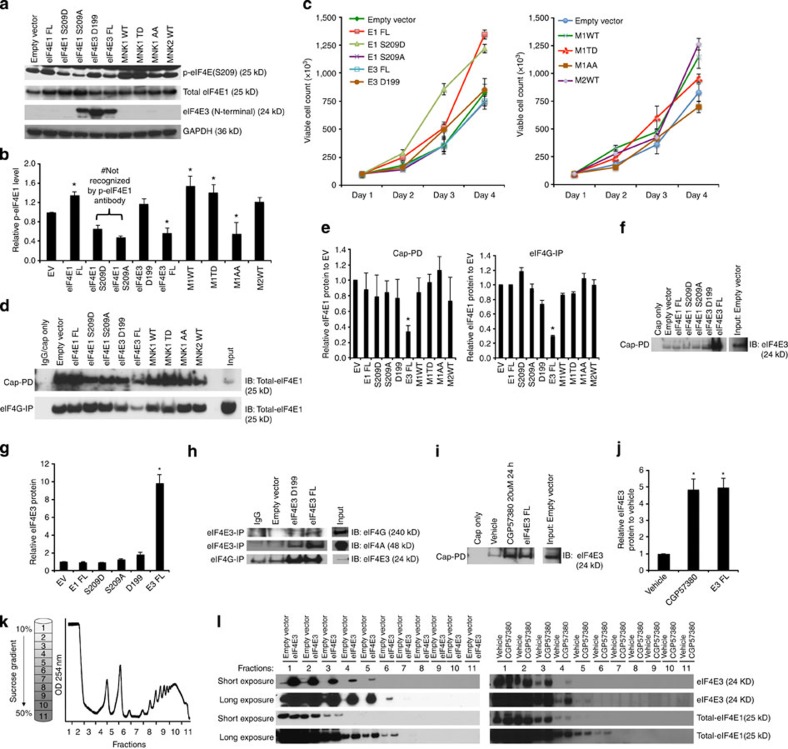
MNK kinase inhibition promotes the activity of an eIF4E3-defined eIF4F-3 translational complex. (**a**) Western blot showing total and phospho-eIF4E1(S209) and total eIF4E3 in stable eIF4E1, eIF4E3 and MNK wildtype and mutant cell lines generated from HLY-1 parent cells. Note: eIF4E3-D199 mutant is detectable only with the N-terminal eIF4E3 antibody. FL=full length, S209D=phosphomimetic, S209A=phosphonull, D199=C-terminal truncation. (**b**) Densitometry analysis showing relative phospho-eIF4E1(S209) levels of eIF4E and MNK mutants as in Fig. 5a. Mean±s.e.m., *n*=3, *P-value of Student’s *t*-test <0.05. Note: a p-eIF4E1 antibody does not detect S209D or S209A mutations (**c**) Trypan blue exclusion assay showing growth curve of eIF4E mutants (left panel) and MNK mutants (right panel) shown in Fig. 5a. Values are mean±s.e.m., *n*=3, *P-value of Student’s *t*-test <0.05. (**d**) Stable HLY-1 cells expressing wild-type and mutant eIF4E1, eIF4E3 and MNKs were used for cap-pulldown and eIF4G-IP and probed for eIF4E1. (**e**) Densitometry analysis of total eIF4E1 after cap-pulldown (left panel) or eIF4G-IP (right panel) as in Fig. 5d normalized to empty vector (mean±s.e.m., *n*=3, *P-value of Student’s *t*-test <0.05). (**f**) Immunoblot following cap-pulldown showing eIF4E3 protein bound to cap in stable HLY-1 mutant cell lines. (**g**) Densitometry analysis of total eIF4E3 as in Fig. 5f, mean±s.e.m., *n*=3, *P-value of Student’s *t*-test <0.05. (**h**) Western blot following reciprocal IP in empty vector, eIF4E3 c-terminal truncated mutant (D199) and eIF4E3 wild type (FL) expressing HLY-1 cells (eIF4E3-IP probed for eIF4EG and eIF4A; eIF4G-IP probed for eIF4E3). (**i**) Western blot following cap-pulldown after vehicle (DMSO) or CGP57380 treatment for 24 h of wildtype and untreated eIF4E3 cells (**j**) Densitometry analysis of eIF4E3 in HLY-1 cells as in Fig. 5i, normalized to vehicle-treated wild-type cells. Values are mean±s.e.m., *n*=3, *P-value of Student’s *t*-test <0.05. (**k**) Cartoon illustrating polysomal fractionation by sucrose density gradient separation used in RNA and protein isolation for translatome analysis. (**l**) Western blot of sucrose density gradient fractions (**k**) probed for eIF4E3 and eIF4E1 (short and long exposure) of empty vector and eIF4E3 expressing HLY-1 mutant cells (left) and vehicle or CGP57380 treated cells (right). Blot shown is a representative of three independent experiments. Full immunoblots are shown in Supplementary Fig. 10.

To understand eIF4E1 and eIF4E3 modulation by MNK in DLBCL, we established stable cell lines of HLY-1 (ABC-DLBCL) and Pfeiffer (GCB-DLBCL) expressing wild-type and mutant eIF4E1, eIF4E3 and MNKs. The eIF4E1-S209D is a phosphomimetic mutation while the eIF4E1-S209A is a phosphonull mutation representing constitutively phosphorylated eIF4E1 and phosphorylation-deficient eIF4E1 proteins, respectively, as previously described^19^,^28^,^31^,^32^. It is important to note that the commercial phospho-specific antibody of eIF4E1 does not recognize the S209D mutant^32^; thus, in these experiments we also employed MCL-1 protein levels as a downstream effector readout (MCL-1 being tightly regulated by phospho-eIF4E1). The phosphorylation of eIF4E1 at S209 was enhanced by the enforced expression of wild-type eIF4E1, MNK1, MNK2 and MNK1-TD mutant (a constitutively activated MNK1 mutant) in both HLY-1 and Pfeiffer (Fig. 5a,b and Supplementary Fig. 6). Consistently these cells also exhibited proliferative advantage compared with vector control HLY-1 cells (Fig. 5c). Remarkably, the enforced expression of the eIF4E1-S209A mutant, which cannot be phosphorylated at S209, showed increased expression of eIF4E3 as well as reduced eIF4E1 cap binding compared with vector control and wild-type eIF4E1 (Fig. 5a,d,e). Conventional dogma suggests that the phosphorylation status of eIF4E1 at S209 is solely determined by MNKs; here we find that the abundance of unphosphorylated eIF4E1 is a trigger for eIF4E3 upregulation. By comparing the MNK kinase inhibitor study (Fig. 4a) and the enforced eIF4E1-S209A study (Fig. 5a), we postulate that MNK kinase inhibition modulating dephosphorylated eIF4E1 is the prompt for eIF4E3 upregulation via translation enhancement. To buttress these findings, we repeated these experiments in another cell line (Pfeiffer) and observed similar results (Supplementary Fig. 6). eIF4E3 expression is not very abundant, and its level in a series of cells are shown in Supplementary Fig. 7a. Enforced expression of the MNK1-AA mutant that cannot phosphorylate eIF4E1 also enhanced endogenous eIF4E3 level (Supplementary Fig. 7b).

eIF4E3 has been recently shown to be able to bind m^7^G-cap in an atypical manner, with about 40-fold lower affinity than eIF4E1, using biophysical determinants^7^. It has been hypothesized that eIF4E3 may compete for the same mRNA transcript as eIF4E1, thus displacing eIF4E1 from cap and reducing eIF4E1-cap-mediated translation. Some translational targets of eIF4E1 such as cyclin D1 and VEGF have been shown to be suppressed by enforced eIF4E3 expression^7^. First, to determine the mechanism by which eIF4E3 may reduce eIF4E1 binding to m^7^G-cap, we expressed an eIF4E3 delta199 (D199) mutant with C-terminal truncation that lacks significant cap-binding activity in HLY-1 and Pfeiffer cells. The expression of wild-type eIF4E3 significantly reduced eIF4E1 cap binding, while the eIF4E3-D199 mutant expression did not exert the same effect (Fig. 5d). These findings were consistent when we repeated similar experiments to analyse eIF4E1 binding to the eIF4G complex (Fig. 5d lower panel). Overexpression of wild-type eIF4E3 not only reduced eIF4E1 binding to cap, but also exhibited enhanced binding of eIF4E3 to cap as demonstrated in the cap-pull down experiments (Fig. 5f,g). Taken together, these data strongly confirm that eIF4E3 requires the cap-binding ability to inhibit eIF4E1 from binding cap, potentially via a substrate competition mechanism. As binding to cap alone may not necessarily indicate effective mRNA translation, we next carried out reciprocal immunoprecipitation of eIF4E3 and eIF4G to ask whether eIF4E3 is able to bind the scaffolding protein eIF4G. We found that eIF4E3 was indeed able to bind eIF4G, further enforcing that eIF4E3 may be a functional translation protein, in addition to being an eIF4E1 inhibitory molecule. eIF4E3 also physically associated with eIF4A, another component of the cap-binding complex, necessary for translation initiation (Fig. 5h). We initially showed that eIF4E1 cap-binding was significantly reduced when cells were treated with an MNK inhibitor CGP57380. We next asked whether eIF4E3 cap-binding is enhanced under the same circumstances. We treated cells with an MNK inhibitor, performed cap-pull down assay and found that, in addition to reducing eIF4E1 cap-binding (Fig. 4d), MNK inhibitor CGP57380 enhanced eIF4E3 binding to cap (Fig. 5i). To interrogate the presence of eIF4E3 in the translation initiation complex, we performed sucrose density gradient fractionation (Fig. 5k). Similar to eIF4E1, eIF4E3 was present in the lighter sucrose fractions of the gradient, further affirming its role in translation initiation (Fig. 5l). We also find that MNK inhibitor treatment enhanced the presence of eIF4E3 in these fractions (Fig. 5l).

### eIF4E1 and eIF4E3 exhibit distinct translatome pattern

**Figure 6:**
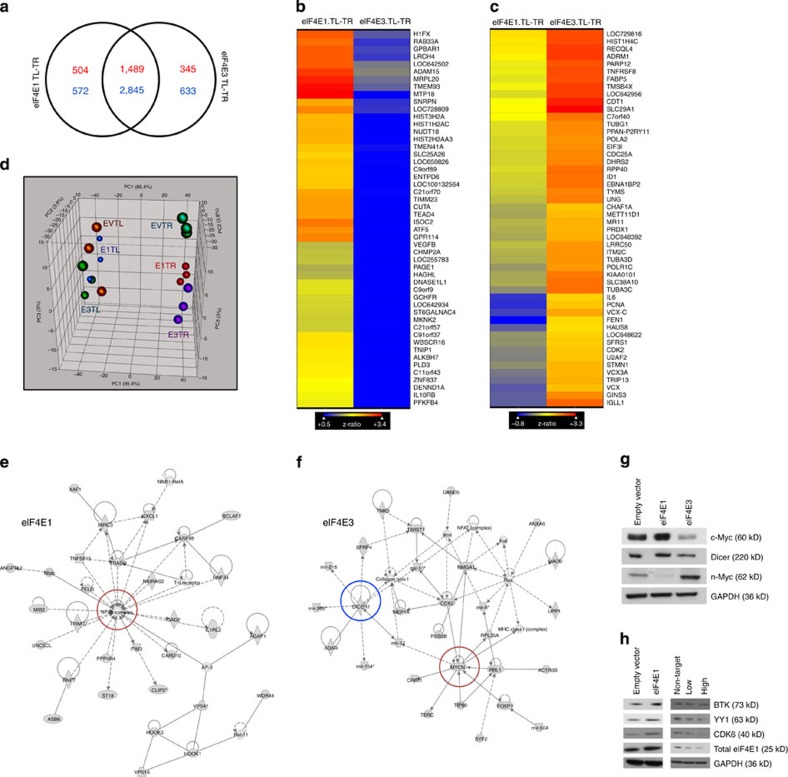
Gene expression analysis of eIF4E1 and eIF4E3 translatome. (**a**) Venn diagram illustrating the distribution of gene targets, which are either mutually or exclusively regulated by eIF4E1 and eIF4E3. (**b**,**c**) Heatmap depicting 50 top gene targets regulated by eIF4E1 (**b**) and eIF4E3 (**c**). Values depicted are *Z*-ratios of expression fold change in the translatome (TL) after eliminating changes in transcriptome (TR) in comparison with vector expressing HLY-1 cells that is, TL-TR data sets. *Z*-ratio values are as depicted in the colour scale shown. (**d**) Principal component analysis showing data distribution of each data subset that is, eIF4E1-translatome (E1TL, blue), eIF4E3-translatome (E3TL, green), empty vector-translatome (EVTL, brown), eIF4E1-transcriptome (E1TR, red), eIF4E3-transcriptome (E3TR, purple) and empty vector-transcriptome (EVTR, turquoise). (**e**) IPA core molecular network analysis of significantly upregulated genes in eIF4E1 translatome exhibiting molecular network interactions that focused on NF-κB complex as a major node. (**f**) IPA core analysis of significantly upregulated genes in eIF4E3 translatome displayed molecular network interactions that showed several diffused nodes leading to downregulation of DICER1 and upregulation of n-Myc. Details of all the bioinformatics analyses performed and the statistical tests used are described in the methods section of Microarray analysis. (**g**) Western blot showing the protein expression of DICER1, n-Myc and c-Myc in vector control, eIF4E1 and eIF4E3 expressing HLY-1 cells. (**h**) Western blot showing three NF-κB targets, YY1, BTK and CDK6 in HLY-1 cells either overexpressing eIF4E1 or treated with eIF4E1 shRNA to knockdown eIF4E1 expression. Full immunoblots are shown in Supplementary Fig. 10.

Our data affirm that eIF4E3 is capable of forming a novel eIF4F cap-binding complex, which we call the eIF4F-3 (‘3’ denoting eIF4E3) via its interaction with eIF4A and eIF4G, and plays a role in the early processes of mRNA translation initiation. We next asked whether eIF4E3 can drive active translation? As the knockdown of eIF4E1 or eIF4E3 caused significant cell death (Supplementary Fig. 8a,b), we overexpressed either protein in HLY-1 cells and performed sucrose density gradient fractionation. RNA extracted from the polysome fractions containing highly translated polysome-bound mRNAs (#9-11, see Fig. 5k) was used for translatome analysis using microarray-chip gene expression analysis. Simultaneously, we obtained total RNA from these cells for transcriptome analysis. Both eIF4E1 and eIF4E3 commonly regulate the translation of a high percentage of genes. Nevertheless, both eIF4E1 and eIF4E3 also displayed distinct gene expression profiles that were significantly altered in the translatome (Fig. 6a–c, Supplementary Table 2). Interestingly, we find MNK2 (*MKNK2*) translation to be regulated by eIF4E1 and translation of targets like *PCNA* and *CDK2* are regulated by eIF4E3. Next, we performed a principal component analysis on the translatome and transcriptome data. Both eIF4E1 and eIF4E3 displayed a mostly overlapping translatome; however, these smaller-by-scale changes at the translatome level resulted in a larger distinction at the transcription level, evidenced by the distinct clustering of each transcriptome data set (Fig. 6d).

By using Ingenuity Pathway Analysis (IPA), we analysed the differentially expressed genes in eIF4E1 and eIF4E3 complete translatomes. IPA Core Analysis revealed NF-κB complex activation as the primary molecular network enriched in eIF4E1 translatome (Fig. 6e). This gives eIF4E1 a prominent role in oncogenic transformation via an NF-κB-dependent transcriptional upregulation in addition to its known role for selecting weak messages (mRNAs that contain long and highly structured untranslatable regions at their 5′-end) for translation^33^. Analysis of the eIF4E3 translatome revealed an important role for eIF4E3 in modulating microRNA maturation via the regulation of dicer. eIF4E3 also affects ADAR (adenosine deaminase, RNA-specific), which is known to regulate RNA editing and transcript stability^34^,^35^,^36^, as well as transcription factors like *n-Myc*, *HMGA1*, *CDX2* and *TWIST1* (Fig. 6f). We corroborated our translatome analysis by western blotting. eIF4E1 but not eIF4E3 cells enhanced c-Myc, a known target of NF-κB transcription activation^37^. Similarly, eIF4E3 cells but not eIF4E1 cells exhibited enhanced n-Myc expression and reduced DICER1 expression (Fig. 6g). To further explore the potential for eIF4E1 driven NF-κB complex activation, we selected three known targets of NF-κB, that is, BTK, YY1 and CDK6, which showed clear correlation with eIF4E1 expression or knockdown in HLY-1 cells (Fig. 6h), and probed in eIF4E1 expressing Pfeiffer and GM02184. We observed a modest increase in CDK6 expression in GM02184, otherwise all other targets were not upregulated by eIF4E1 expression (Supplementary Fig. 9a). Next, to explore whether this phenomenon was restricted to the ABC-DLBCL cells, we knockdown eIF4E1 in another ABC-DLBCL cell line, SUDHL-2, and probed for the same targets. We found that the targets were downregulated, thus showing a strong correlation between eIF4E1 expression and NF-κB targets genes in ABC-DLBCL specifically (Supplementary Fig. 9b).

**Figure 7:**
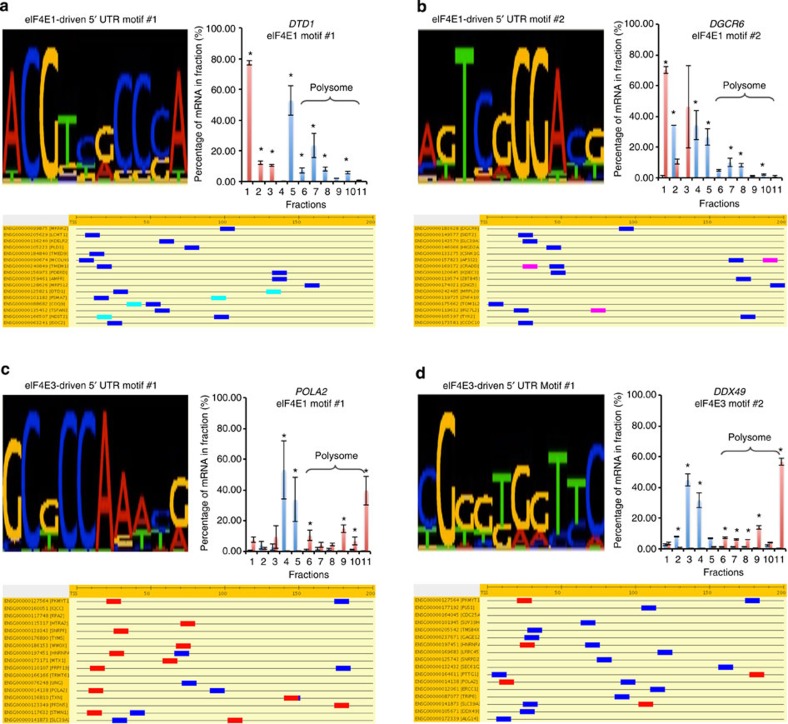
eIF4E1- and eIF4E3-translatome selective 5′-UTR motifs. (**a**) eIF4E1-driven 5′-UTR motif sequence #1, with motif location map illustrating the location of motif #1 (blue) and #2 (cyan) in the 5′-UTR region and RT-qPCR validation of target genes representing motif #1 target, *DTD1*. mRNA percentage was normalized to exogenous luciferase control mRNA level (eIF4E1 in blue and eIF4E3 in red). Values shown represent mean±s.e.m., *n*=3, *P-value of Student’s *t*-test <0.05. (**b**) eIF4E1-driven 5′-UTR motif sequence #2, with motif location map illustrating the location of motif #2 (blue) and #1 (pink) in the 5′-UTR region and RT-qPCR validation of target genes representing motif #2 target, *DGCR6*. mRNA percentage was normalized to exogenous luciferase control mRNA level (eIF4E1 in blue and eIF4E3 in red). Values shown represent mean±s.e.m., *n*=3, *P-value of Student’s *t*-test <0.05. (**c**) eIF4E3-driven 5′-UTR motif sequence #1, with motif location map illustrating the location of motif #1 (red) and #2 (blue) in the 5′-UTR region and RT-qPCR validation of target genes representing motif #1 target, *POLA2*. mRNA percentage was normalized to exogenous luciferase control mRNA level (eIF4E1 in blue and eIF4E3 in red). Values shown represent mean±s.e.m., *n*=3, *P-value of Student’s *t*-test <0.05. (**d**) eIF4E3-driven 5′-UTR motif sequence #2, with motif location map illustrating the location of motif #2 (blue) and #1 (red) in the 5′-UTR region and RT-qPCR validation of target genes representing motif #2 target, *DDX49*. mRNA percentage was normalized to exogenous luciferase control mRNA level (eIF4E1 in blue and eIF4E3 in red). Values shown represent mean±s.e.m., *n*=3, *P-value of student *t*-test <0.05.

We identified two most significantly enriched 5′-untranslated region (UTR) motifs in eIF4E1 and eIF4E3 translatome (Fig. 7). The locations of the motifs in their corresponding targets are illustrated in the location map charts. We adapted a luciferase-normalized RT-qPCR approach employed by the Sabatini lab^38^ to measure the percentage of target mRNA in each polysome fraction to validate the selection of motif-containing transcripts by eIF4E1 or eIF4E3 (Fig. 7). Transcripts containing eIF4E3-driven motifs, that is, *POLA2* and *DDX49,* were most abundant in the heavier fractions of eIF4E3 cells, while the same transcripts were displaced to the lighter fractions in the eIF4E1 cells. Similarly, transcripts containing the eIF4E1-driven motifs, that is, *DGCR6* and *DTD1,* were displaced to the lighter fractions in the eIF4E3 cells. In eIF4E1 cells, these transcripts were more abundant in the heavier fractions in comparison with eIF4E3 cells; however, more transcripts accumulated in the monosomal and early polysomal fractions. These findings are consistent with the presence of excessive eIF4E1 initiating mRNA translation; however, the cells may still require other factors that may be motif specific and rate-limiting for eIF4E1-driven translation, presenting a potential avenue for future studies. The complete lists of eIF4E1 and eIF4E3-driven targets displaying respective motifs and the nucleotide frequency statistics are shown in Supplementary Tables 3–5. Our analysis comparing previously published mTOR-driven motifs revealed that eIF4E1- and eIF4E3-driven motifs are novel and independent from previously published mTOR-driven motifs^38^,^39^, although mTOR can affect eIF4E1 activity via 4E-BP1 phosphorylation (Supplementary Table 6).

## Discussion

Translation dysregulation can be extended to almost all types of human malignancies and serves as an attractive target for potential therapeutic intervention. Here, we have presented data to support a unique role for MNKs in DLBCL, which serve as a ‘switch’ that modulate eIF4E1- and eIF4E3-driven translation in cells. We have demonstrated that MNK1 and MNK2 are differentially distributed in the GCB and ABC subsets of DLBCL, and that the preferential expression of MNK2 in ABC-DLBCL can be a contributing factor to the aggressive nature of this subtype. MNK2, even at low levels, exhibits high basal activity compared with MNK1 and is not easily manipulated by upstream regulators^14^.

The novel regulation of eIF4E1 level by MNKs illustrates a reciprocal correlation between the two proteins. The regulation of eIF4E1 level by MNK in a kinase-independent fashion is mediated via down regulation of eIF4E1 mRNA translation by lack of MNKs. However, enforced expression of MNKs did not significantly upregulate eIF4E1 expression, suggesting the possibility of other cellular surveillance mechanisms that may work to maintain eIF4E1 at a physiological level.

**Figure 8:**
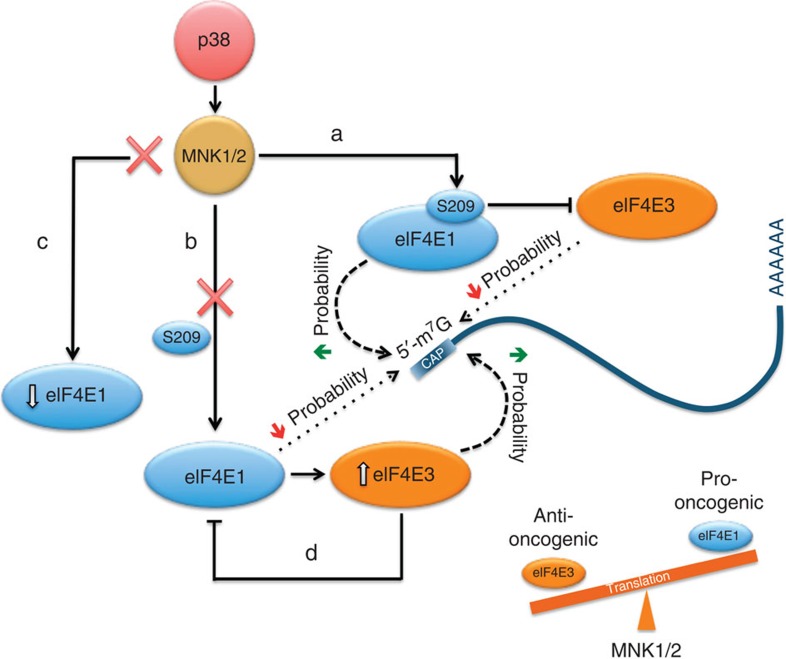
The regulation of eIF4E1 and eIF4E3 by MNKs in DLBCL. (**a**) On activation by p38, MNKs phosphorylate eIF4E1 at S209. (**b**) In the absence of MNK kinase activity, eIF4E1 cannot be phosphorylated, while in the (**c**) absence of MNK protein expression or its physical suppression, eIF4E1 protein expression is downregulated. (**d**) The unphosphorylated eIF4E1 (at S209) form is stimulatory for eIF4E3 protein upregulation. Increased abundance of eIF4E3 in a cellular context enhances the ability for eIF4E3 to bind cap. The relative abundance of either eIF4E1 or eIF4E3 is determined by MNKs. The accessibility to mRNA cap structure by both eIF4Es mandates a distinct cellular translatome that dictates pro- or anti-oncogenic phenotype.

We have shown that the absence of MNK kinase activity on eIF4E1 results in upregulation of eIF4E3, which can attenuate eIF4E1 cap binding (Fig. 8). While inhibition of eIF4E1 is emerging as an attractive target in malignancies, there are potential caveats. As eIF4E1 inhibition may reduce the pro-oncogenic cellular translatome, the compensatory translation by eIF4E3 may, in addition to promoting growth inhibition, also provide a mechanism to sustain cell viability without rapid proliferation. By regulating the translation of crucial transcription factors like n-Myc, eIF4E3 can also potentially upregulate the transcription of many pro-proliferative targets, which may contribute to the relapse of tumours treated with MNK or eIF4E1 inhibitors.

We first identified upregulation of eIF4E3 when we inhibited MNK kinase activity. Our subsequent investigation revealed that the lack of phosphorylation in eIF4E1 at S209, the effector of MNK kinase inhibition, was the trigger for eIF4E3 upregulation. It was not previously known that eIF4E3 could initiate effective mRNA translation, although its *in vitro* cap-binding ability has been recently described^7^. Here, we show that eIF4E3 binds to cap and forms a novel eIF4F cap-binding complex. eIF4E3 not only exerts inhibitory effects on eIF4E1-mediated translation, but also facilitates translation of select messages. We find eIF4E1-mediated translation enhances the NF-κB pathway, a well-recognized pathway essential for lymphocyte development, proliferation and survival, which if deregulated is linked to T- and B-cell lymphomagenesis^40^,^41^. Hariri *et al.*^42^ have reported that eIF4E1 is a direct transcriptional target of NF-κB and aberrant expression of NF-κB-driven eIF4E1 was evident in a subset of acute myeloid leukaemia. In this study, we find that the inverse is also true, where NF-κB complex activation is indirectly enhanced via eIF4E1-mediated translation.

eIF4E1 and eIF4E3 are able to recognize and bind the m^7^G-cap structure on mRNA. It is unclear how the two proteins can facilitate the selection of mRNA to be translated. One hypothesis is that eIF4E1 and eIF4E3 select for interacting factors, via altering conformational changes of the cap-binding complex or by causing steric hindrance to the assembly of other components of the translation machineries. Nonetheless, both eIF4E1 and eIF4E3 display preferential 5′-UTR motifs in their highly regulated transcripts.

We conclude that MNKs play a pivotal role in the control of eIF4E1 and eIF4E3 levels and function *in vivo,* and this fine balance is crucial to maintain a normal cellular phenotype. Aberrant disruption to this balance, perturbing cap-binding preference for both eIF4E1 or eIF4E3 proteins, enables a switch, causing subsequent alterations in the cellular translatome that translates a transformed phenotype.

## Methods

### Cell lines, culture and generation of stable cell lines

HEK293T/17, SUDHL-2, SUDHL-4, SUDHL-6, Toledo, GM02184, Karpas422, U2932, Jurkat and Pfeiffer cell lines were obtained from American Type Culture Collection. TMD8 and HLY-1 cells were gracious gifts from Dr Lisa Rimsza (University of Arizona, AZ) and Dr Ari Melnick (Weill Cornell, NY), respectively. HCT116 was a kind gift from Dr Rena Lapidus and Brandon Cooper (Translation Core Facility, University of Maryland Baltimore). HEK293T/17 was grown in DMEM supplemented with 10% FBS (Atlanta Biologicals), 100 unit ml^−1^ penicillin and 0.1 mg ml^−1^ streptomycin (Sigma-Aldrich) at 37 °C with 5% CO_2_. All other cells were grown in RPMI-1640 media supplemented with 10% FBS, 100 unit ml^−1^ penicillin and 0.1 mg ml^−1^ streptomycin at 37 °C with 5% CO_2._ In experiments where cells were treated with select inhibitors, exponentially growing cells were plated at 2.5 × 10^5^ cells per ml density before treatment and incubated for variable time points at 37 °C with 5% CO_2_, before harvesting for further analysis. Stable cell lines of HLY-1 and Pfeiffer overexpressing eIF4E1, eIF4E1-S209D, eIF4E1-S209A, eIF4E3, MNK1, MNK2, MNK1-TD and MNK1-AA proteins were generated by retroviral transduction and were selected and maintained with puromycin (2 μg ml^−1^).

### Plasmids, lentiviral production and transduction

Expression plasmids for eIF4E1-S209D and eIF4E1-S209A were a gracious gift from Dr Hans-Guido Wendel (Memorial Sloan Kettering Cancer Center, NY). Expression plasmids for eIF4E1, eIF4E3 and eIF4E3-D199 were cloned from previously described constructs^7^. MNK1, MNK2, MNK1-TD and MNK1-AA were a gracious gift from Dr Herman Gramm (Novartis Institutes for BioMedical Research, Switzerland). All expression plasmids were cloned into a lentiviral-vector system CD513B (System Biosciences, CA). For lentiviral packaging, HEK293T/17 cells were seeded at 40% confluence and transfected the following day with psPAX2 and pMD2.G (Addgene plasmids 12,260 and 12,259; deposited by Dr Didier Trono). Virus-containing medium was concentrated using Amicon Ultra-15 100 kDA centrifugal filters (EMD Millipore) as per the manufacturer’s instruction. HLY-1 and Pfeiffer cells (2x10^5^ cells per ml density in a 10 ml volume) were treated with 4 μg ml^−1^ of polybrene (American Bioanalytical) and centrifuged for 35 mins at 800 r.c.f. at 30 °C. Cell pellets were resuspended in virus-containing medium for 12 h. Cells were selected and maintained with puromycin (2 μg ml^−1^).

### Gene knockdown using shRNA

Lentiviral-shRNA plasmids against MNK1 and MNK2 were purchased from Sigma-Aldrich (MNK1: TRCN0000314869, MNK2: TRCN0000342226 and TRCN0000006098, designed and validated by The RNAi Consortium shRNA library, Broad Institute of Harvard and MIT). Transduction viruses were prepared as described above and the multiplicity of infection (MOI) of all shRNA determined in HEK293T/17. Cells were transduced via spinoculation as described above. Following transduction, cells were harvested at 24 or 48 h for analysis. All shRNA sequences are listed in Supplementary Table 7.

### Chemicals

MNK inhibitors CGP57380 and cercosporamide were purchased from Tocris and Sigma-Aldrich, respectively. VX702 (p38 inhibitor) was purchased from Cayman Chemical, and MEK inhibitor AZD6244 was purchased from CalBiochem. All chemicals were dissolved in DMSO before treating cells in culture to a final DMSO concentration not exceeding 0.1%. Solutions were prepared fresh on the day of experiment.

### Western blotting

Cells were lysed in lysis buffer (25 mM Tris pH 8.0, 125 mM NaCl, 1 mM MgCl_2_, 1% Triton X-100, 1 mM sodium orthovanadate, 1 mM sodium fluoride, 1X protease inhibitor (Sigma-Aldrich), phosphatase inhibitor cocktails #2 and #3 (Sigma-Aldrich), and 1 mM PMSF). Cell lysates were resolved on a Nupage 4–12% Bis-Tris gradient gel (Life Technologies) and probed using antibodies recognizing phospho- and total eIF4E1 (Cell Signaling #9741 and Santa Cruz sc-9976), phospho- and total ERK (#9101 and #9102, Cell Signaling), c-Myc (#9402), n-Myc (#9405), p-MNK1/2 (#2111), dicer (#3363), eIF4A (#2013) (Cell Signaling), GAPDH (ab8245), BTK (ab54219), YY1 (ab12132) (Abcam), eIF4E3 (Proteintech: N-terminal, #17282-1-AP), MNK1 (sc-6965), MNK2 (sc-6964), CDK6 (sc-7961), eIF4G (sc-11373), MCL-1 (#sc-819) (Santa Cruz) or GFP (EVN-AB513, Axxora). All primary antibodies were used at 1:1,000 dilution. Densitometry analyses were performed using ImageJ software (NIH) and presented as ratio of target band signal intensity to GAPDH band signal intensity. Full immunoblots are presented in Supplementary Fig. 10.

### Immunoprecipitation and cap analogue pull down

For immunoprecipitation (IP) experiments, 500 μg of cell lysate was incubated with a primary antibody (2 μg eIF4G antibody or 13.6 μg eIF4E3 antibody) overnight at 4 °C before preclearing with protein A Sepharose beads (40 μl slurry, 2.5 h, 4 °C). Lysate-bead mixture was washed three times in IP buffer (50 mM Tris–HCL pH 7.5, 150 mM NaCl, 1 mM EDTA, 1 mM EGTA, 1% Triton X-100, 0.5% Nonidet-40, 1 mM sodium orthovanadate, 1 mM sodium fluoride, 1X protease inhibitor (Sigma-Aldrich), phosphatase inhibitor cocktails #2 and #3 (Sigma-Aldrich), and 1 mM PMSF) followed by addition of 4 × loading buffer. For cap analogue-pull down (Cap-PD), 250 μg of cell lysate was incubated with 50% m^7^GTP-Agarose bead slurry (0.5 ml, overnight, 4 °C, Jena Bioscience AC-141S), followed by three washes in IP buffer and diluted in 4 × loading buffer. Samples were boiled before loading on 4–12% Bis-Tris protein gels.

### Flow cytometry

For cell cycle analysis, transduced cells were collected (4 × 10^5^ cells) by centrifugation (500 r.c.f., 5 min, 4 °C), and washed twice with cold PBS, followed by overnight fixation in 75% ethanol at 4 °C. Cells were stained with propidium iodide (PI) at 50 μg ml^−1^ in 0.1% BSA and analyzed within 1 h of staining on BD FACSCanto. For cell proliferation assay, 10 million cells were washed twice in PBS and resuspended in 0.5 ml PBS. Carboxyfluorescein succinimidyl ester (CFSE) was prepared at 10 μM (2 × ) concentration in PBS. CFSE 2 × solution (0.5 ml) was added drop wise into the cell suspension while rapidly mixing the cells on vortex. Cells were incubated at 37 °C for 10 mins in the dark with occasional mixing. Cold RPMI medium with 10% FBS was added to stop CFSE incorporation. Cells were pelleted and resuspended in medium with or without drug treatment. After 48 and 72 h, cells were pelleted and resuspended in 0.5% BSA/PBS solution. TO-PRO was used to gate out dead cells. All flow cytometry analysis was run on BD FACSCanto and data analysed with FlowJo software (Tree Star Inc., OR).

### Polysome (Polyribosome) fractionation

A total of 1 × 10^7^ cells were harvested using polysome extraction buffer (150 mM KOAc, 2.5 mM Mg(OAc)_2_, 20 mM K-N-2-hydroxyethylpiperazine-*N*′-2-ethanesulfonic acid, pH. 7.5, RNaseOUT, and protease inhibitor cocktail). Lysates were fractionated using a linear sucrose gradient (10-50% sucrose) in a Beckman Coulter SW41 swinging bucket rotor (35,000 r.p.m., 3 h, 4 °C). Heavy fractions (#9, 10 and 11, see Fig. 5k) were isolated for RNA extraction using Trizol.

### RNA extraction and real-time PCR

Exponentially growing cells were harvested by centrifugation. Total RNA was extracted using Trizol and reverse transcribed using Superscript III (Invitrogen) for standard real-time PCR (RT–qPCR) analysis. For polysomal fraction RT–qPCR analysis, RNA was extracted from each sucrose fraction by standard Trizol method. Five nanograms of polyA+ synthetic luciferase mRNA (Promega) was spiked into the RNA of each fraction for normalization (following methods established by Thoreen *et al.*^38^). All RNA samples were reverse transcribed using Superscript III. RNA from tissue microarray (TMA) slides containing paraffin sections pre-classified as ABC- and GCB-DLBCL by an independent pathologist^26^ was isolated using RecoverAll Total Nucleic Acid Isolation Kit for FFPE (Life Technologies) according to the manufacturer’s protocol^25^. RT-qPCR reactions were carried out using SYBR green (Quanta) detection system on a Bio-Rad CFX Connect equipment. All primer sequences used are tabulated in Supplementary Table 7.

### Bromodeoxyuridine (BrdU) ELISA

We performed BrdU incorporation assay using BrdU Cell Proliferation Assay kit (Cell Signaling). In brief, cells were incubated with labelling medium containing BrdU for 4 h at 37 °C and 5% CO_2_. Cells were then fixed and processed for BrdU detection following the manufacturer’s protocol.

### Immunohistochemistry staining

We performed immunohistochemistry (IHC) staining of primary lymphoma tissues on a tissue microarray (TMA) slide (US Biomax, LM801) for p-eIF4E1 staining at Mass Histology Services (Boston, MA). In brief, TMA slide was de-waxed and hydrated. Slide was then pre-treated with citrate buffer and rinsed in water. Slide was treated with 3% H_2_O_2_ followed by another rinse in water and two washes in PBS. Sample was then blocked in 2% horse serum and incubated in a primary antibody at 1:250 dilution for 1 h at room temperature. After three washes, the slide was incubated with an ImmPRESS HRP conjugated secondary antibody (Vector Labs) for 45 min at room tenperature. Following two more washes in PBS, slide was treated with DAB substrate and rinsed in water. Slide was then counterstained with haematoxylin and differentiated in acid alcohol (2 quick dips). Finally, the slide was placed under running water for 10 mins, dehydrated and mounted with coverslip.

### Microarray analysis

RNA from total (for transcriptome analysis) or polysomal fractions (#9,10 and 11, see Fig. 5k; for translatome analysis) from empty vector and eIF4E1- or eIF4E3-expressing cells from three independent experiments was extracted and labelled using Illumina Total Prep RNA Amplification Kit (Ambion). Illumina Human HT-12 v4 expression microarray BeadChips containing 47,000 RefSeq transcripts (Illumina) were used for microarray analysis. Data were filtered by detection (*P*≤0.02), then normalized by *Z*-score transformation and tested for significant differences in signal intensity. Data quality was analysed by scatter plot, principal component analysis and *Z*-score-based hierarchy clustering. ANOVA test was performed to exclude genes with large variance. Genes that were significantly altered with a *Z*-ratio of above 1.5 (fold increase) or below -1.5 (fold decrease) display a false discovery rate, *FDR*≤0.3 (using empirical Bayes approach) and *P*-value <0.05 in comparison with control samples were considered statistically significant. To filter gene sets that are solely altered in the translatome without changes in transcriptome, genes that were significantly altered in the translatome were screened and eliminated if concurrent changes in transcriptome were observed. Overlapping genes between two groups were analysed and presented in a Venn diagram. Genes that showed statistically significant differential expression in the translatome of each study group (eIF4E1 and eIF4E3) versus control group were subsequently analysed using IPA ( www.analysis.ingenuity.com; Ingenuity Systems). IPA core analysis was performed to identify top molecular functions involved in eIF4E1 and eIF4E3 translatome. Network analysis uses a curated knowledge based on known functional interactions and protein functions to algorithmically infer biochemical interactions.

### Motif enrichment analysis

To discover the enrichment of motifs in the 5′-UTR regions of the targets of eIF4E1 and eIF4E3, we utilized the AMADEUS motif discovery platform^43^. The 5′-UTR sequences for the human gene set were downloaded from the resources available on the AMADEUS website. The genes that were found to be part of the eIF4E1 and eIF4E3 translatomes were used as the target gene sets and compared with the human gene set. The background and target gene sets were tested for GC and length bias. Motif discovery was performed to identify 10 bp motifs localized within a range 200 bp downstream of the transcription start site (TSS). The enrichment factor and *P*-values were computed using the hypergeometric (HG) over-representation scores for each identified motif. The motifs were also tested for strand bias, chromosomal preference and localization. The position-weighted matrix of candidate motifs were further compared with that of the TOP/TOP-like motif described by Thoreen *et al.* and Eliseeva *et al.*^38^,^44^

### Statistical analysis

Where applicable, a two-tailed Student’s *t*-test was performed; values were considered statistically significant at *P*-value **<**0.05. All experiments were performed in at least three biological replicates for statistical analysis.

## Additional information

**Accession codes:** Complete microarrayarray results have been deposited in Gene Expression Omnibus (GEO), NCBI, with the following accession number, GSE61691.

**How to cite this article:** Landon, A. L. *et al.* MNKs act as a regulatory switch for eIF4E1 and eIF4E3 driven mRNA translation in DLBCL. *Nat. Commun.* 5:5413 doi: 10.1038/ncomms6413 (2014).

## Supplementary Material

Supplementary Information Supplementary Figures 1-10, Supplementary Tables 1-7.
