# Supplementary material for: MNKs act as a regulatory switch for eIF4E1 and eIF4E3 driven mRNA translation in DLBCL
Source: Nat Commun. 2014 Nov 18;5:5413. doi: 10.1038/ncomms6413 (PMC4238046; doi:10.1038/ncomms6413)
Supplement: Supplementary Information — Supplementary Figures 1-10, Supplementary Tables 1-7. [file ncomms6413-s1.pdf]

Supplemental Figure 1

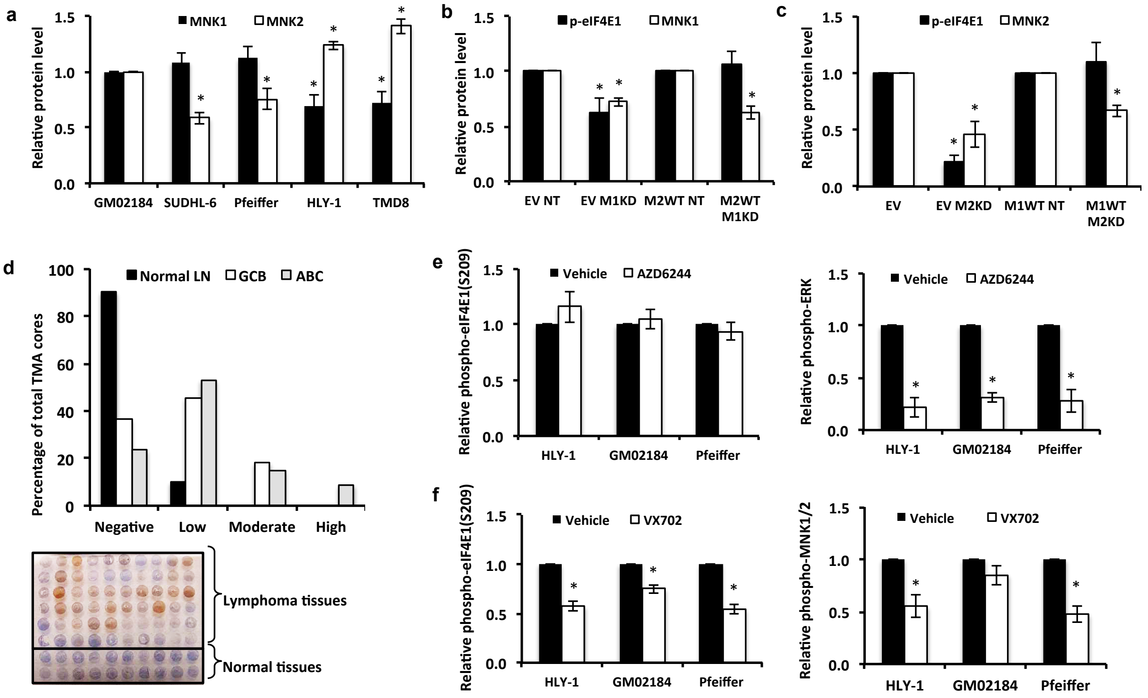

Sup. Fig. 1. Densitometry and IHC analysis. (a) Densitometry analysis showing relative band intensity of MNK1 and MNK2 to GAPDH from immunoblot in Fig.1b. (b) Densitometry analysis showing relative band intensity of p-eIF4E1 (S209) and MNK1 from immunoblot in Fig.1g. (c) Densitometry analysis showing relative band intensity of p-eIF4E1 (S209) and MNK2 from immunoblot in Fig.1h. (d) Top: Bar graph showing percentage of tissue microarray (TMA) cores stained for p-eIF4E1 level in normal lymph node (LN), ABC-DLBCL and GCB-DLBCL samples. Bottom: Broad field photographic image illustrating p-eIF4E1 IHC staining in TMA slides with normal and lymphoma sample mapping. (e) Densitometry analysis showing relative band intensity of phospho-eIF4E1 (S209) and phospho-ERK to GAPDH in HLY-1 cells from immunoblot in Fig.2a. (f) Densitometry analysis showing relative band intensity of phospho-eIF4E1 (S209) and phospho-MNK1/2 to GAPDH in HLY-1 cells from immunoblot in Fig.2b. Values are mean of three independent measurements (mean ± S.D., **p*-value of student *t*-test < 0.05).

Supplemental Figure 2

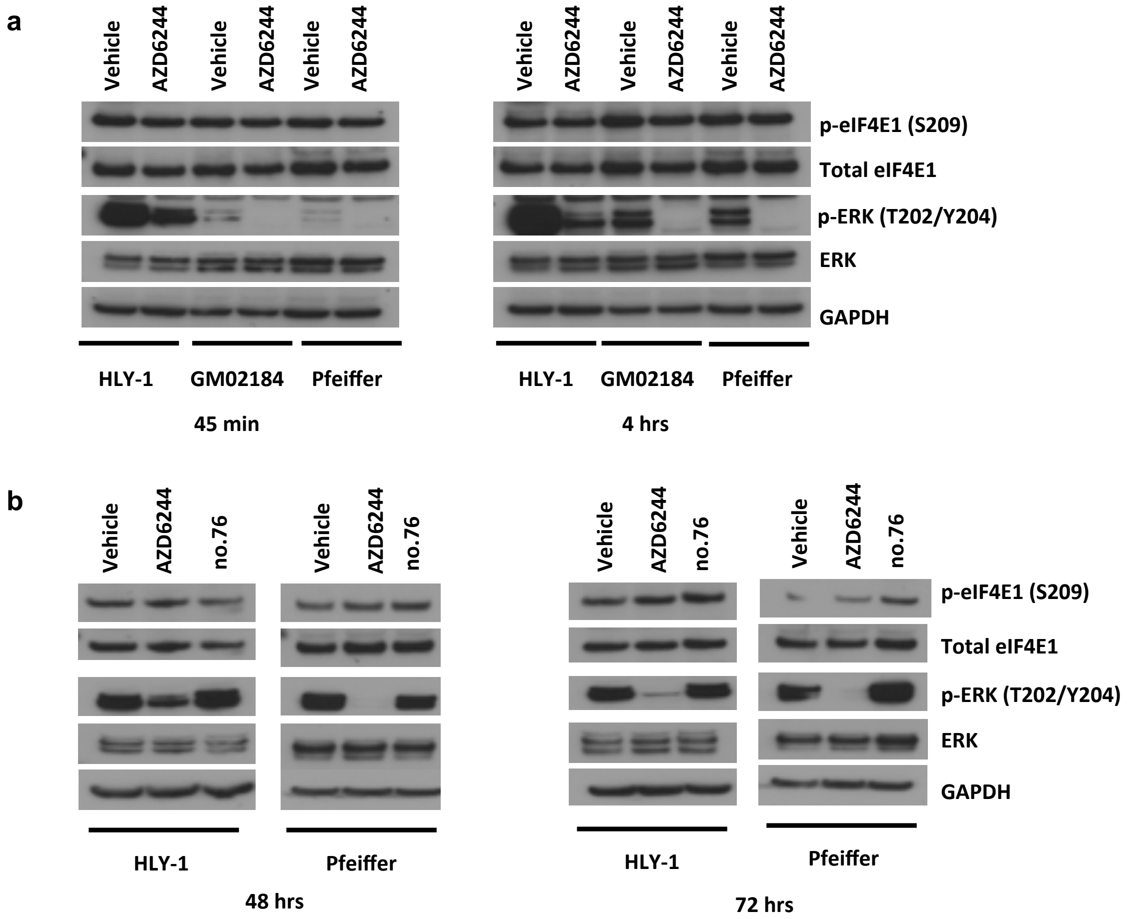

Sup. Fig. 2. MEK and ERK inhibition does not impact eIF4E1 phosphorylation. (a) Western blot of cell lysates from HLY-1, GM02184 and Pfeiffer cell lines treated with 200 nM of MEK inhibitor, AZD6244 for 45 min or 4 hrs, and probed for total and phospho-eIF4E1, total and phospho-ERK and GAPDH. (b) Western blot showing cell lysates from HLY-1 and Pfeiffer cell lines treated with 200 nM of MEK inhibitor or 20 μM of ERK2 inhibitor, no.76 for 48 or 72 hrs, and probed for total and phospho-eIF4E1, total and phospho-ERK and GAPDH. All blots shown are representative of at least three experimental replicates.

Supplemental Figure 3

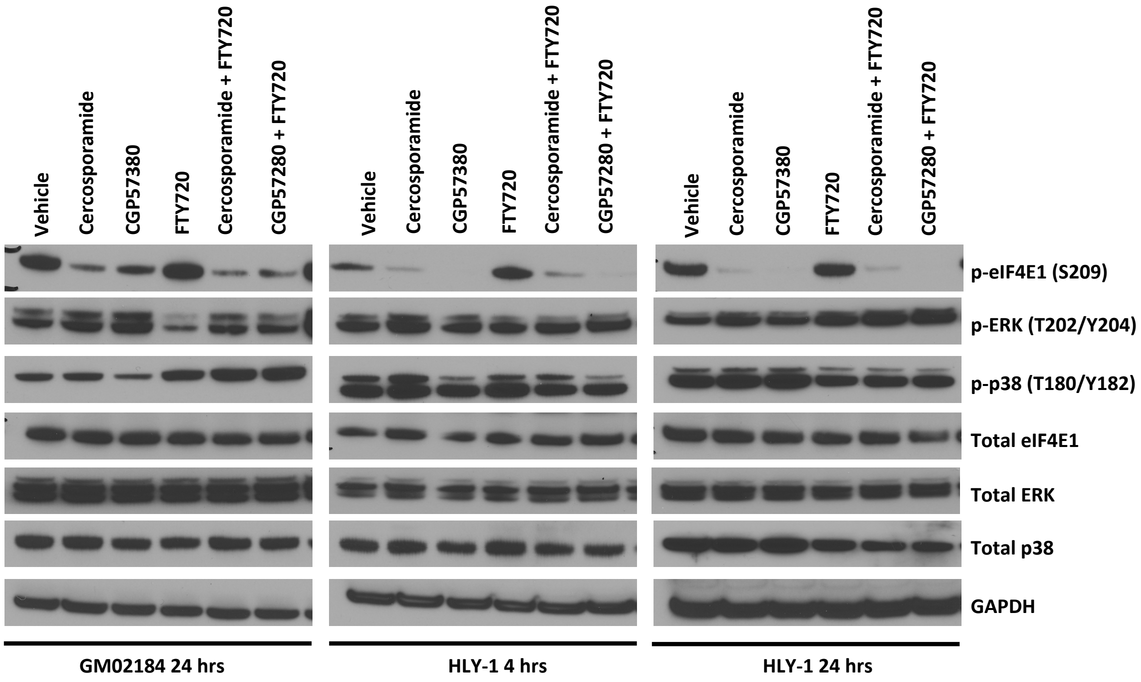

Sup. Fig.3. **PP2A activation using FTY720 did not affect eIF4E1 phosphorylation**. Western blot of cell lysates from GM02184 and HLY-1 cells treated with MNK inhibitors; CGP57380 (20 μM) or Cercosporamide (5 μM), PP2A activator, FTY720 (20 μM) or in combination as indicated for 4 or 24 hrs, and probed for total and phospho-eIF4E1, total and phospho-ERK, total and phospho-p38 and GAPDH. All blots shown are representative of at least three experimental replicates.

Supplemental Figure 4

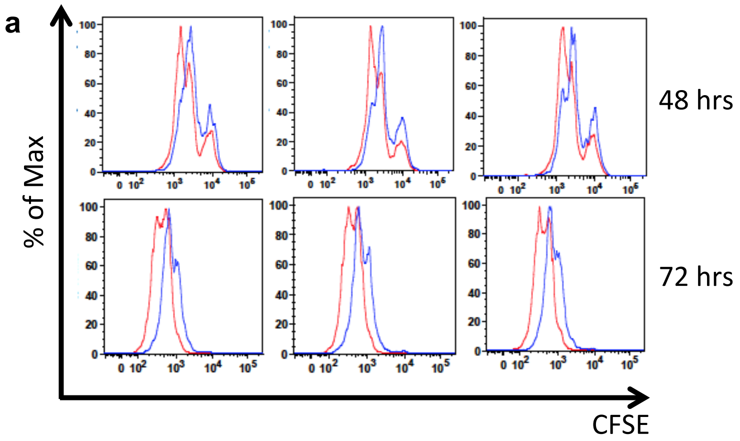

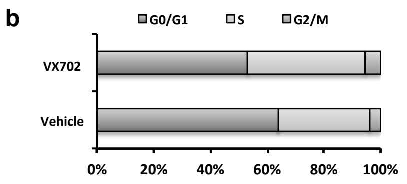

Treatment	G0/G1	S	G2/M
Vehicle	63.4±0.7	31.9±0.9	3.81±0.1
VX702	52.7±0.4	41.3±2.7	5.41±1.3

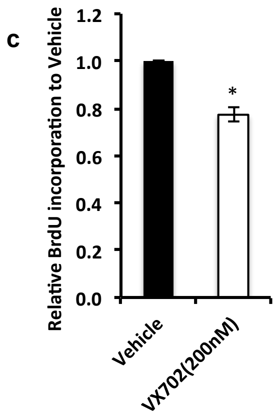

Sup. Fig.4. **Effect of p38 inhibition on DLBCL cell line** (a) CFSE cell proliferation assay at 48 and 72 hrs post DMSO (-) or VX702 (200 nM) (-) treatment in triplicates (one representative figure is shown in Fig.2g). (b) Cell cycle analysis of HLY-1 cells after 72 hrs treatment with either vehicle or VX702 (200 nM), showing a marginal increase in S-phase cell populations. Values are represented as percent of total cell population (mean ± S.D., n=3). (c) BrdU incorporation ELISA assay of HLY-1 cells treated with vehicle (■) or VX702, 200 nM (□). Values are representative of three independent experiments (mean ± S.D., * *p*-value of student *t*-test < 0.05).

Supplemental Figure 5

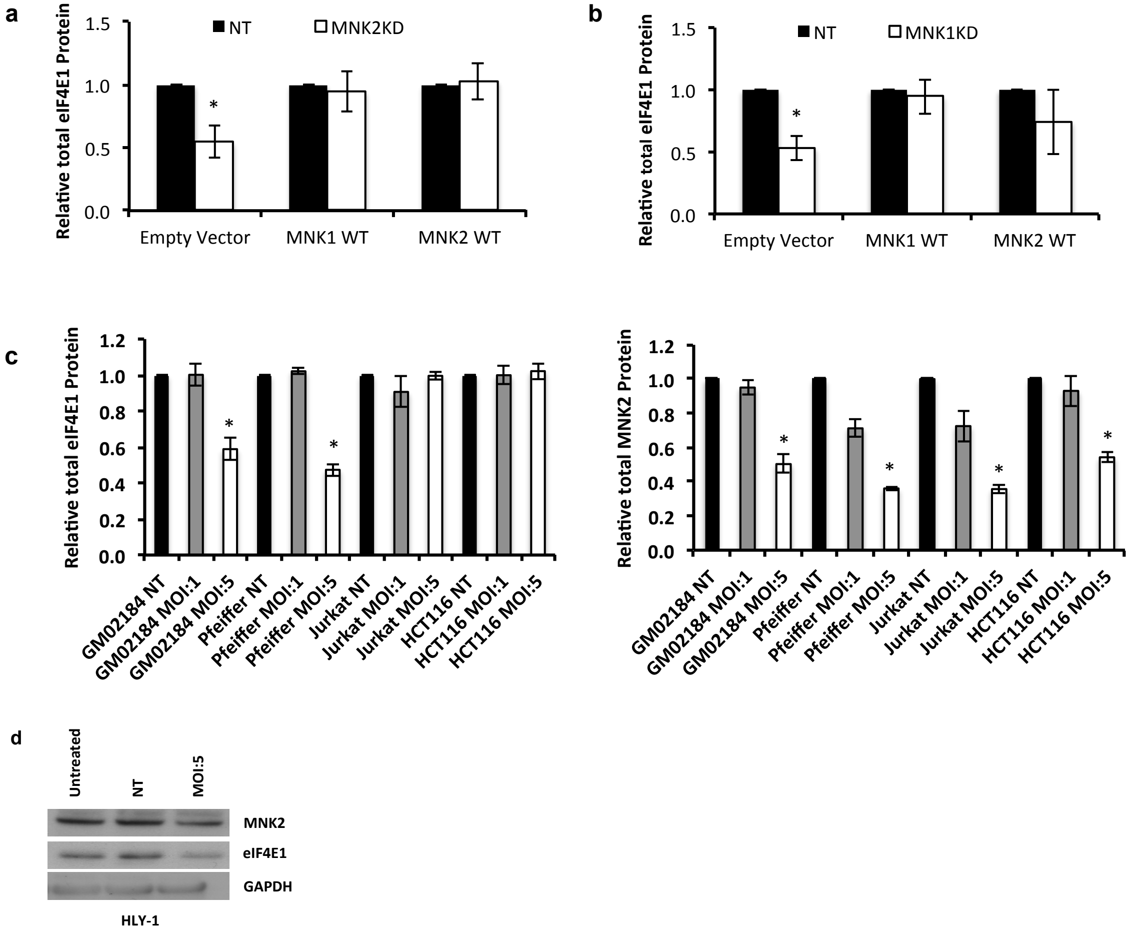

Sup. Fig. 5. Densitometry analysis and eIF4E1 knockdown. (a-b) Densitometry analysis showing relative band intensity to GAPDH of total eIF4E1 in HLY-1 cells treated with either NT (■), (a) MNK2 or (b) MNK1 KD shRNA (□) (MOI=10) from (a) Fig.3k or (b) Fig.3l. (c) Densitometry analysis showing relative band intensity to GAPDH of total eIF4E1 (left) and MNK2 (right) in HLY-1 cells treated with either NT (■), MNK2 KD MOI:1 (■), or MNK2 KD MOI:5 shRNA (□) from Fig.3m. All densitometry values are mean from three independent measurements (mean ± S.D., **p*-value of student *t*-test < 0.05). (d) Western blot analysis of HLY-1 cells untreated, with NT shRNA or MNK2 KD shRNA (TRCN0000006098) and probed for MNK2 and eIF4E1.

Supplemental Figure 6

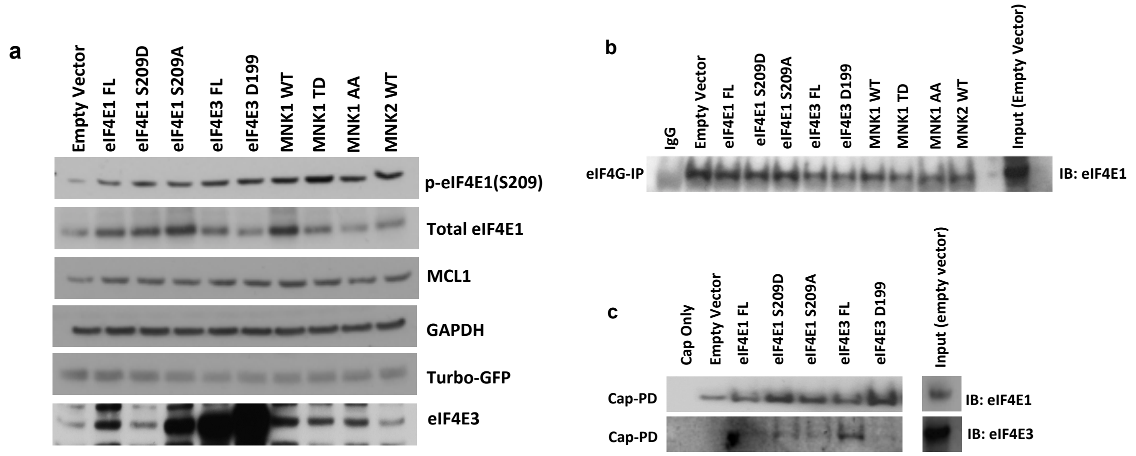

Sup. Fig 6. Analysis of Pfeiffer cell lines stably expressing wildtype or mutated eIF4E1, eIF4E3, MNK1 and MNK2 (a) Western blot analysis of Pfeiffer cells transduced with various constructs as indicated and probed for total and phospho-eIF4E1 (S209), MCL-1, GAPDH, GFP and eIF4E3. (b-c) Immunoblot of Pfeiffer cell lysate after (b) IP using eIF4G antibody or (c) cap pull down, and probed for eIF4E1 or eIF4E3. All blots shown are representative of at least three experimental replicates.

Supplemental Figure 7

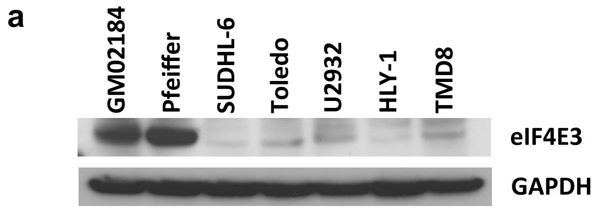

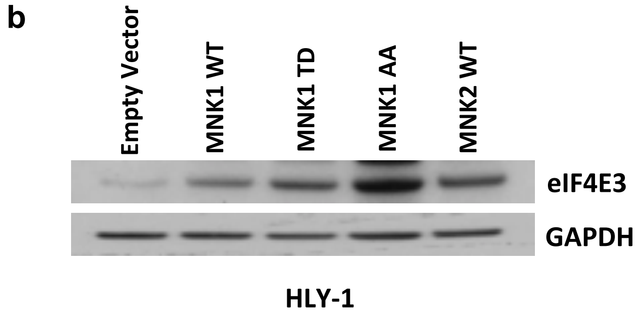

Sup. Fig.7. eIF4E3 expression in various cell lines and HLY-1 cells expressing MNK wildtype and mutant proteins (a) Western blot analysis of various DLBCL cell lines for basal levels of eIF4E3 expression. (b) Western blot analysis of eIF4E3 in HLY-1 mutant cell lines. All blots shown are representative of at least three experimental replicates.

Supplemental Figure 8

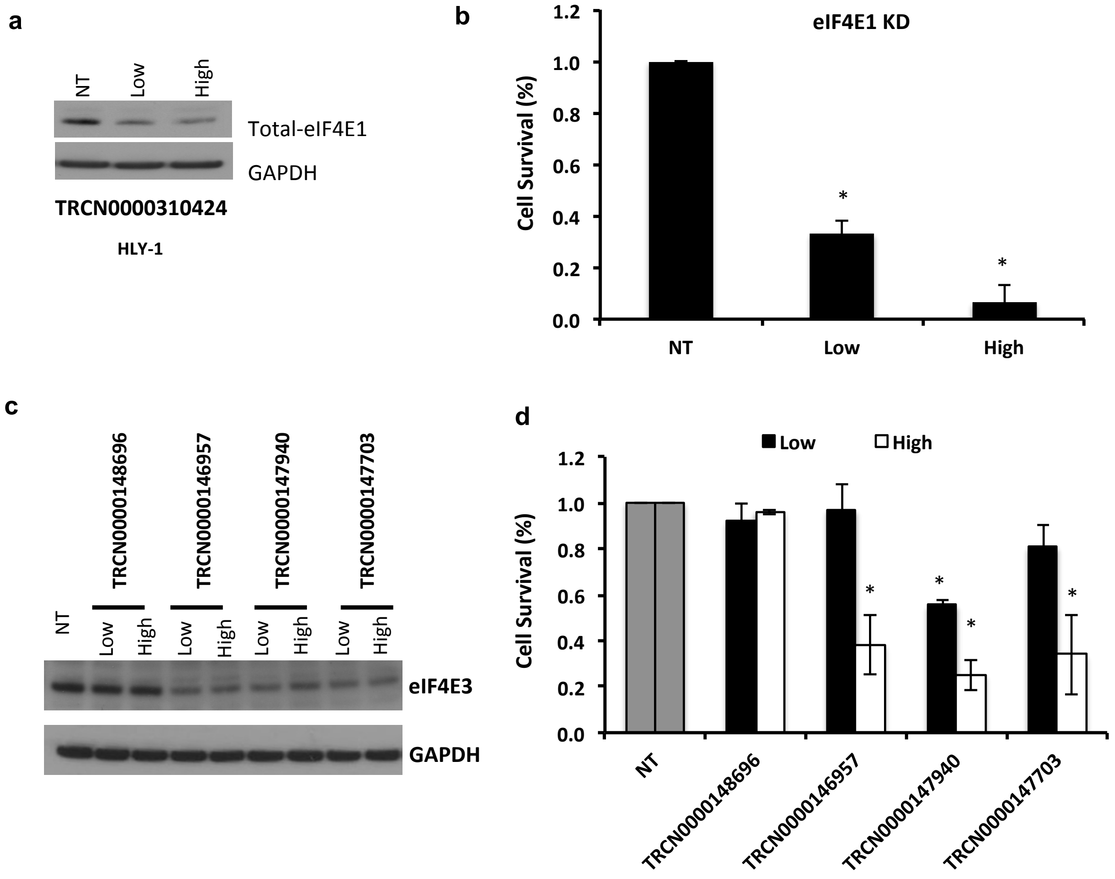

Sup. Fig. 8. shRNA knockdown of eIF4E1 and eIF4E3 (a) Western blot analysis of HLY-1 cells treated with eIF4E1 knockdown (KD) shRNA at 48 hrs post transduction, and probed for eIF4E1 (b) Trypan blue exclusion assay for cell survival of HLY-1 cells with eIF4E1 KD from Sup.Fig.8a. Values are mean ± S.D., n=3 **p*-value of student *t*-test <0.05 (c) Western blot analysis of HLY-1 cells treated with various eIF4E3 KD shRNA as indicated, after 48 hrs post-transduction. (d) Trypan blue exclusion assay for cell survival of HLY-1 cells treat with various eIF4E3 KD shRNAs, 48 hrs post-transduction from Sup. Fig.8c in comparison to NT treated cells (■). Values are mean ± S.D., n=3, **p*-value of student *t*-test < 0.05.

Supplemental Figure 9

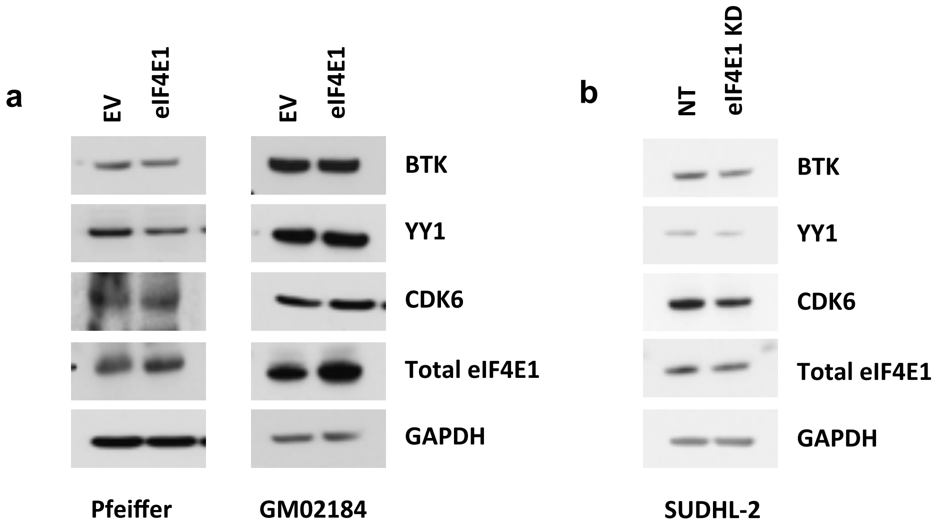

Sup. Fig. 9. NF-κB targets validation. (a) Western blot of Pfeiffer and GM02184 cell lines either expressing empty vector (EV) or eIF4E1, probed for BTK, YY1, CDK6, total eIF4E1 and GAPDH. (b) Western blot of SUDHL-2 (ABC-DLBCL) cell line treated with non-target (NT) or eIF4E1 shRNA for 48 hrs, probed for BTK, YY1, CDK6, total eIF4E1 and GAPDH. All blots shown are representative of at least three experimental replicates.

Supplemental Figure 10

Figure 1b

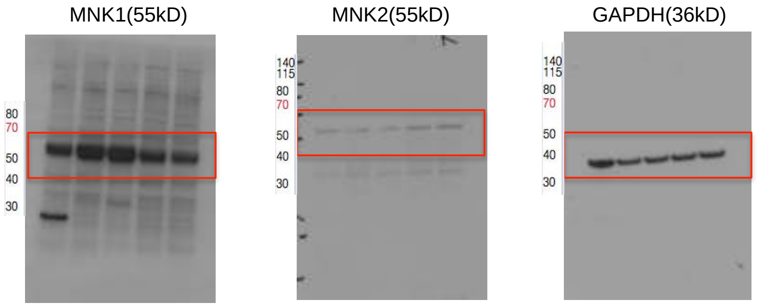

Figure 1h

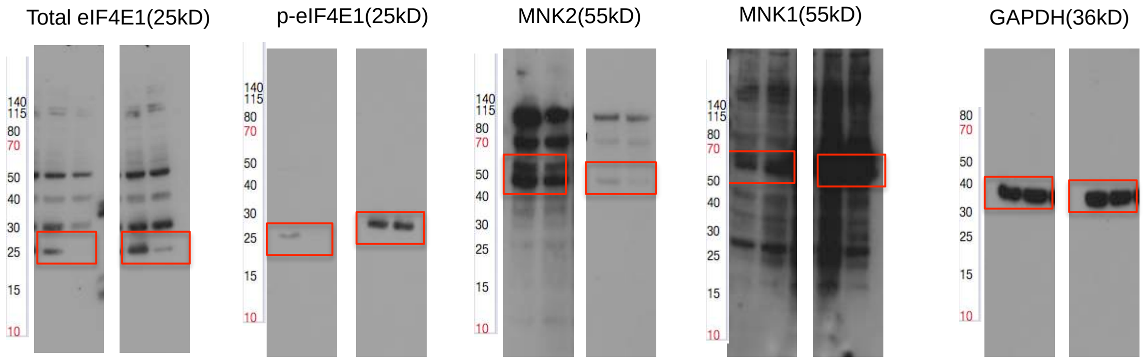

Figure 1g

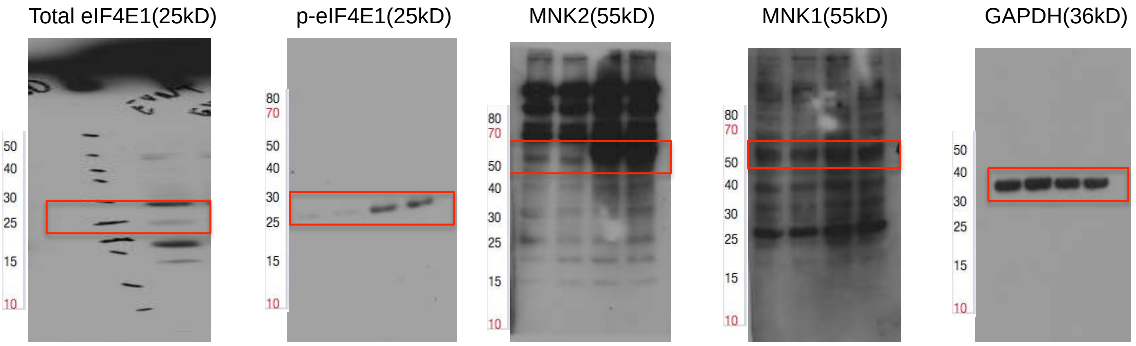

Figure 2a

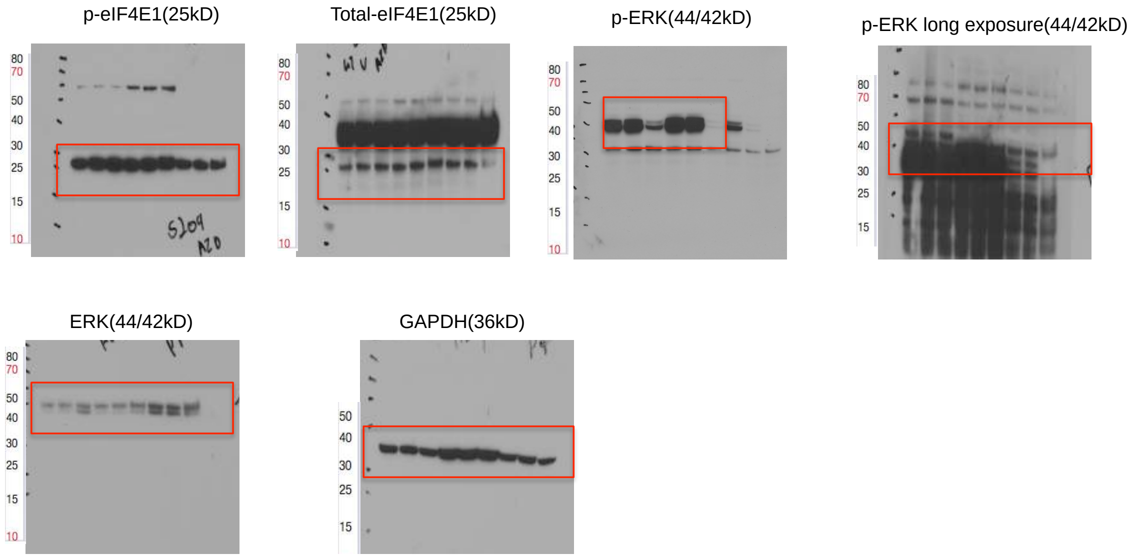

Figure 2b

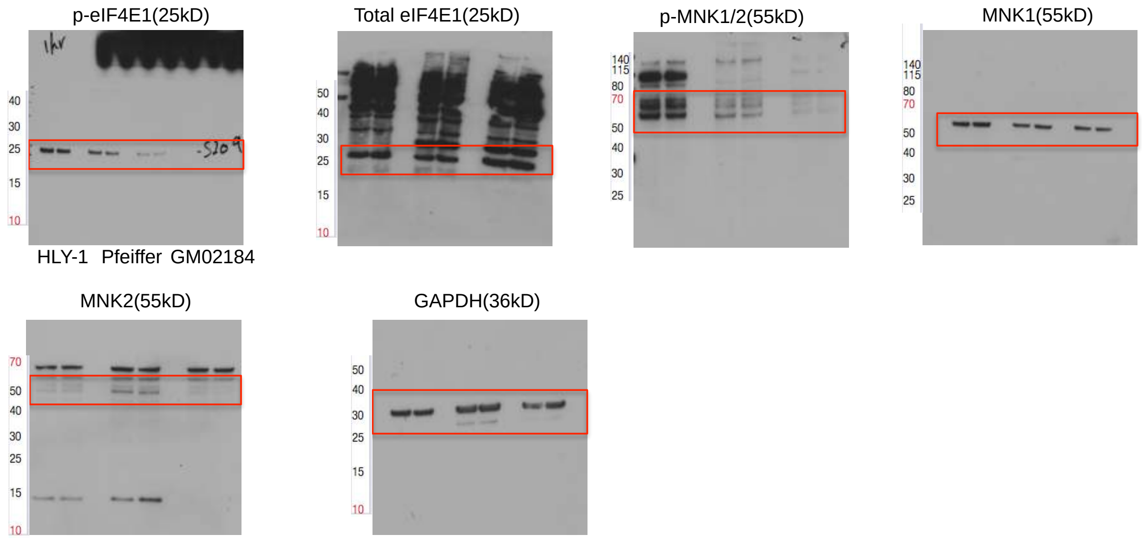

Figure 2c

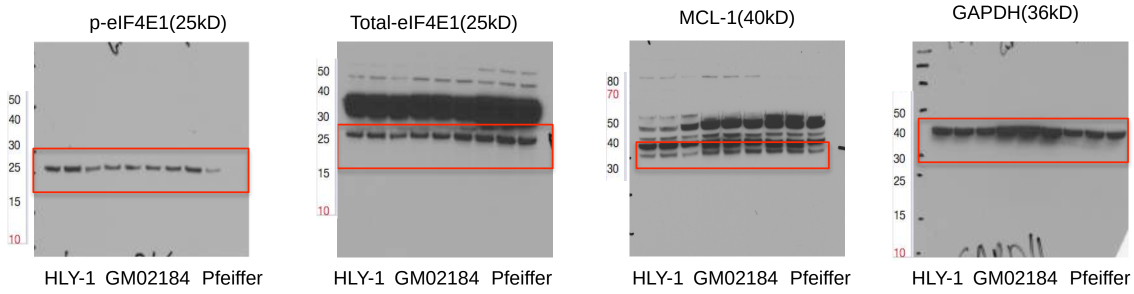

Figure 2h

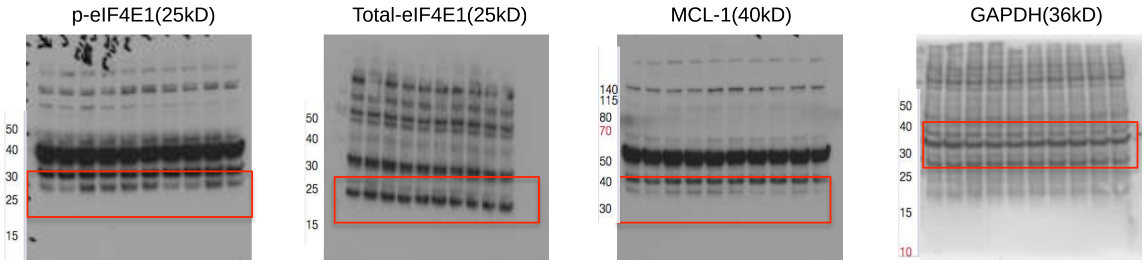

Figure 3a

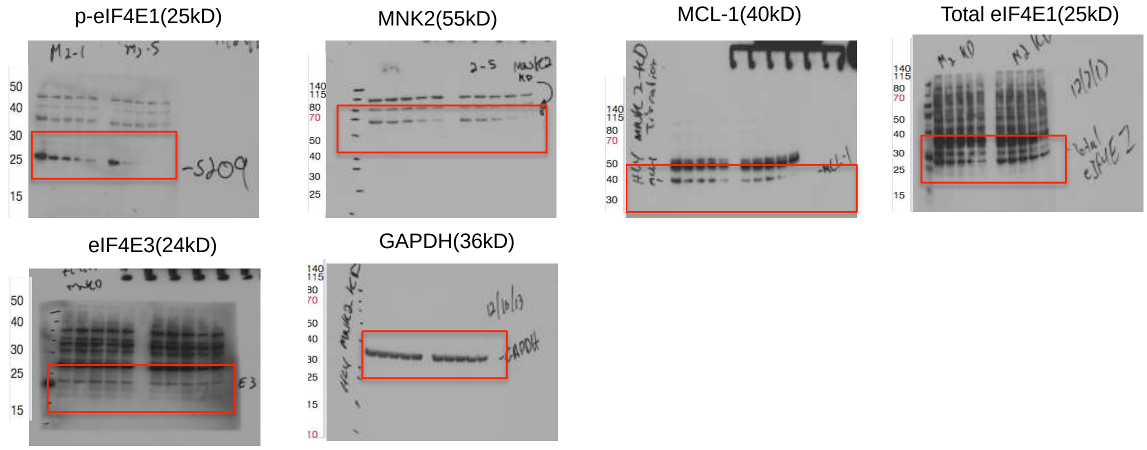

Figure 3f

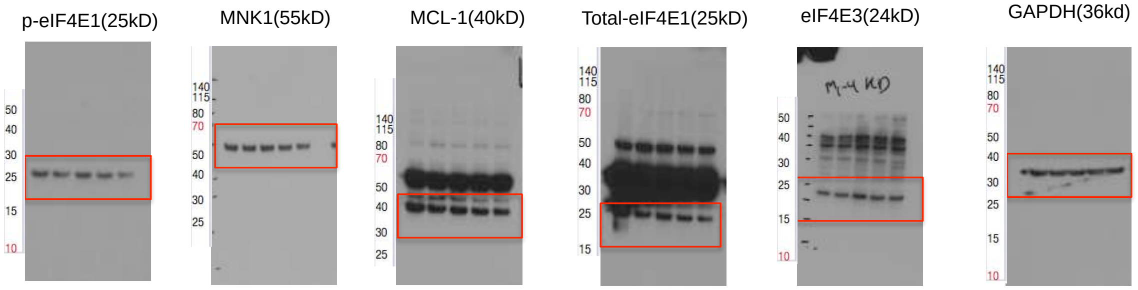

Figure 3k

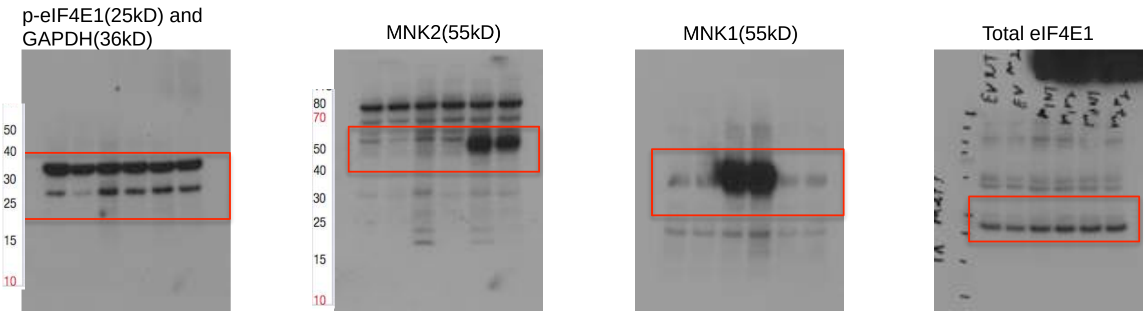

Figure 3i

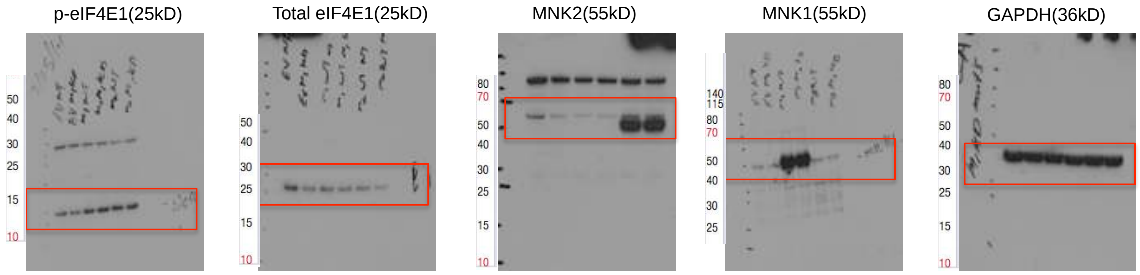

Figure 3n

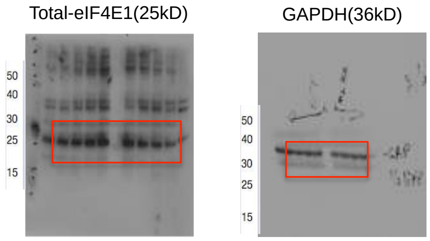

Figure 3m

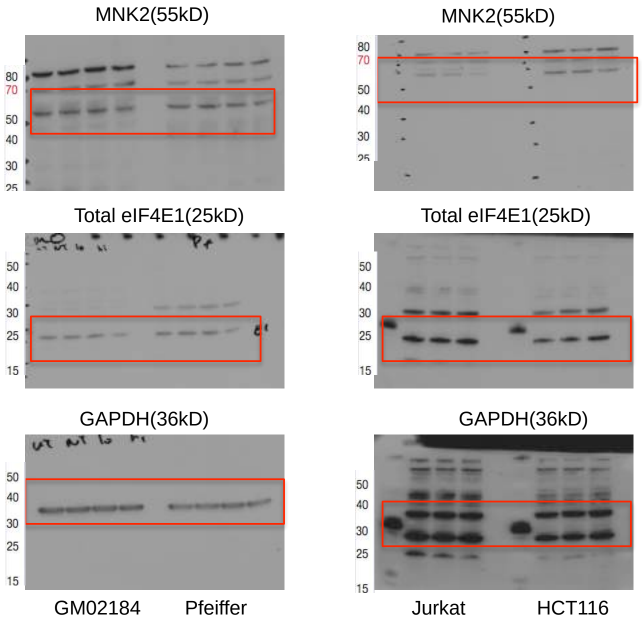

Figure 4a

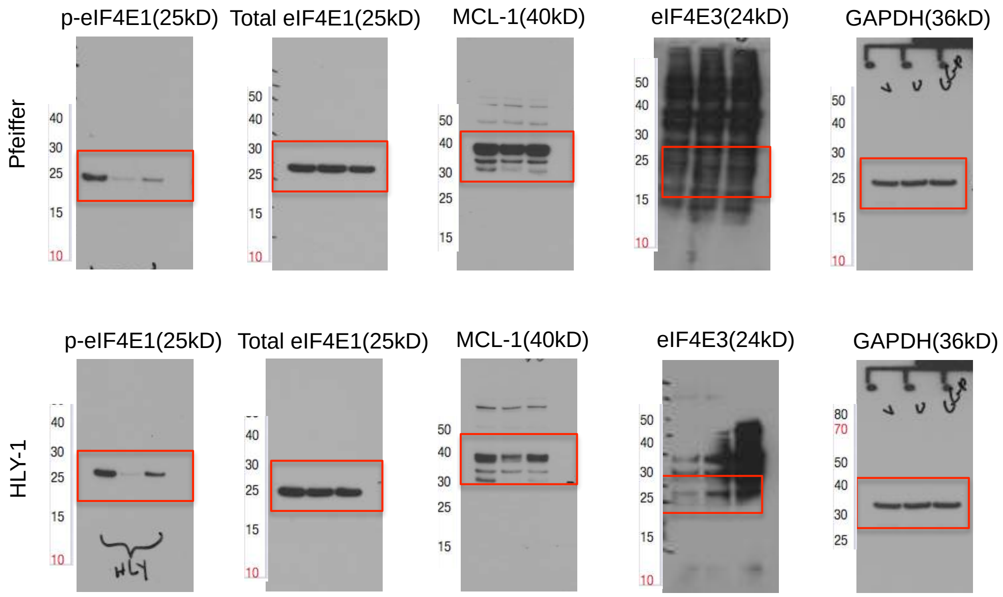

Figure 4d

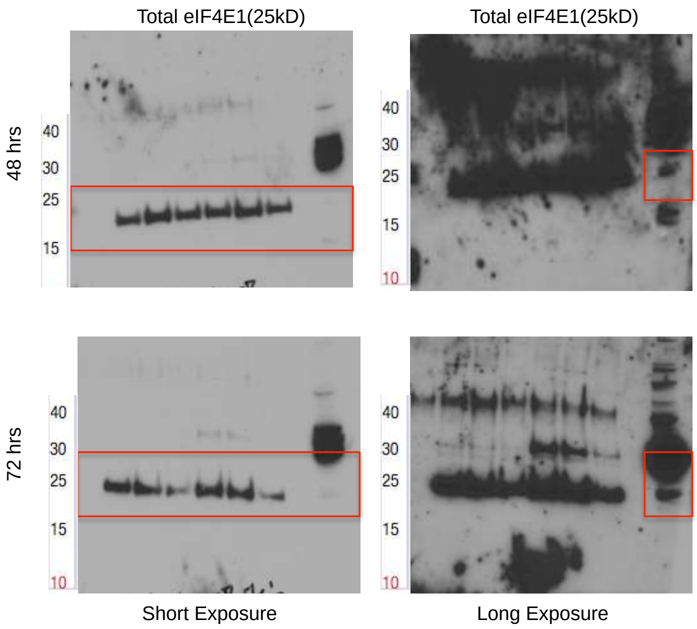

Figure 4g

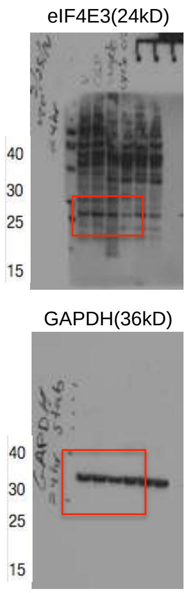

Figure 5a

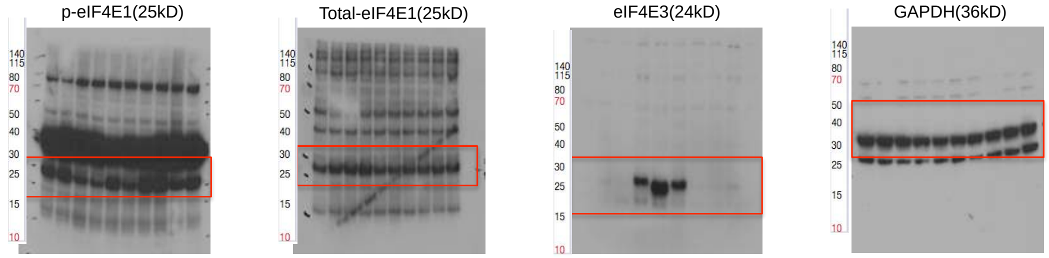

Figure 5d

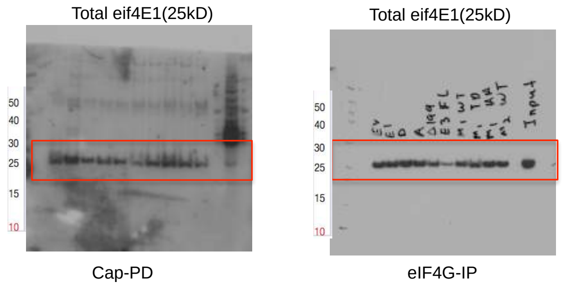

Figure 5f

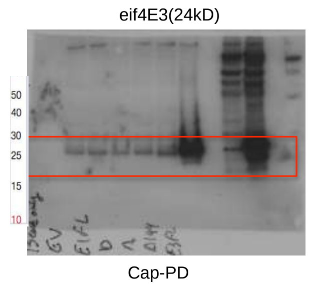

Figure 5h

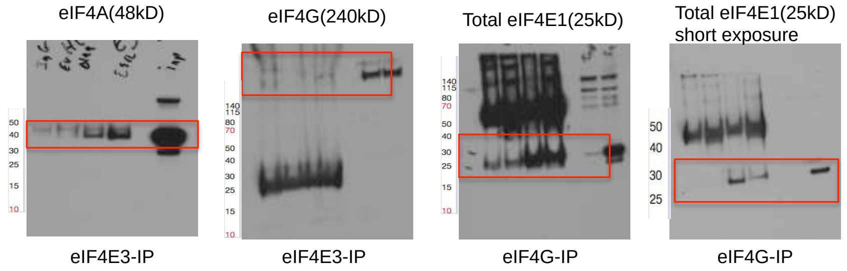

Figure 5i

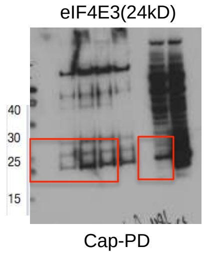

Figure 5l

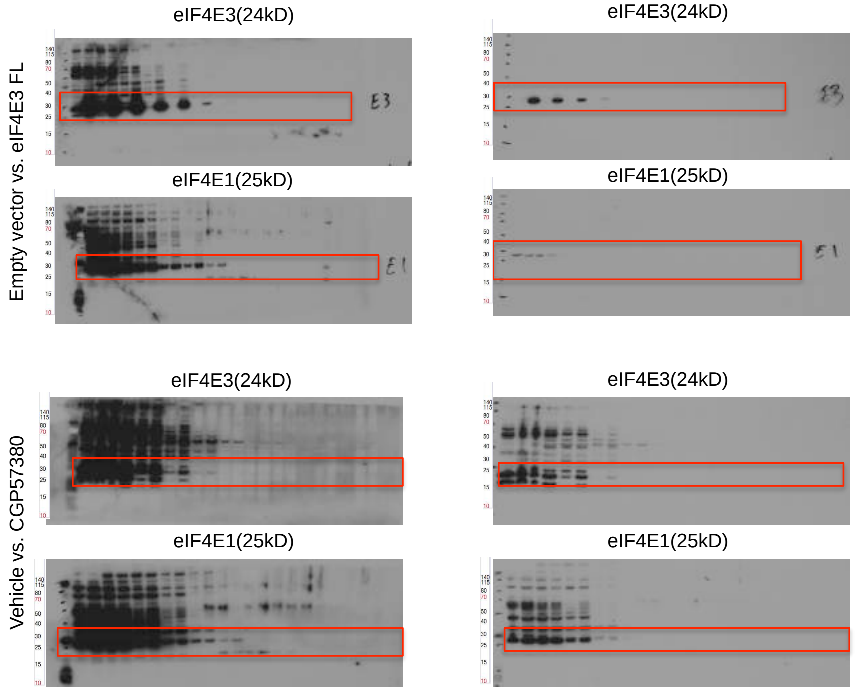

Figure 6g

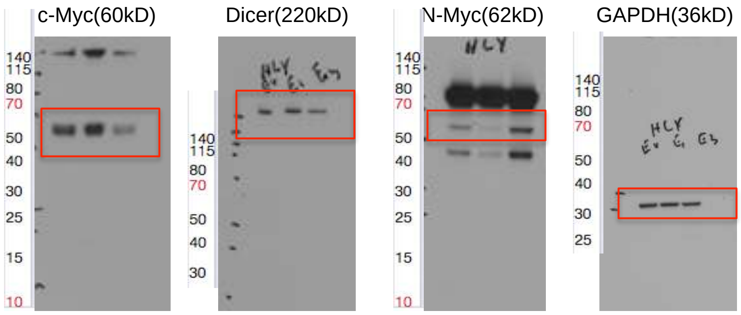

Figure 6h
Empty vector and eIF4E1

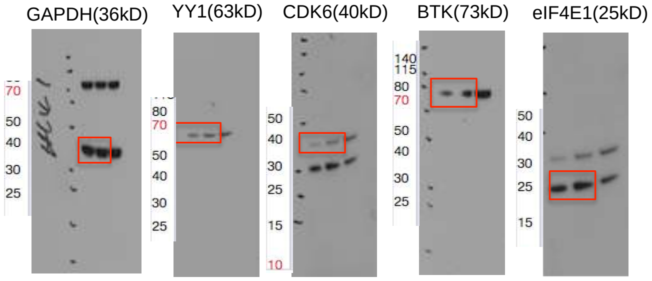

Figure 6h
Non-target and eIF4E1 shRNA

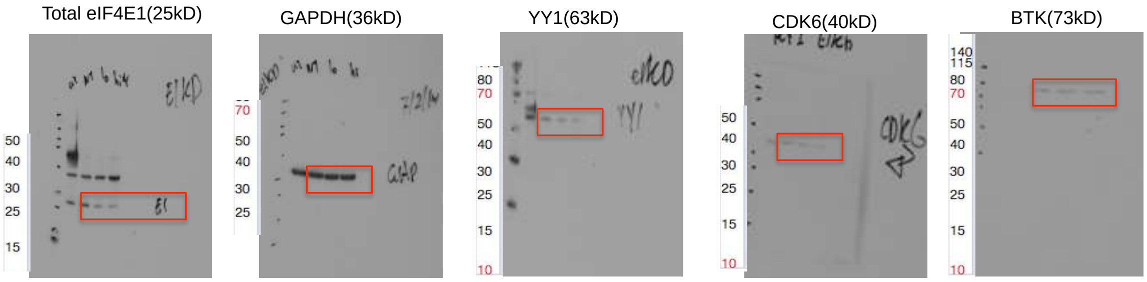

Sup.Fig.10. Full immunoblots of segments shown in the main figures

Supplemental Table 1

Data mining from oncomine (https://www.oncomine.org) showing the distribution of MNKs in DLBCL

MNK2

GCB/ABC classified?	Range of Expression	Sample Number	*Increase/decrease or split	Source
No	Up 1.5-4.5 fold	166	Increase	Hummel et al, NEJM, 2006
No	Up-2+fold	271	Increase	Salaverria et al, Blood, 2011
No	Up 6+fold	40	Increase	Booman et al, J Pathol 2008/10/01
Yes	Up 5+ fold	414	Increase	Lenz et al, NEJM, 2008
No	Up 2.5+ fold	252	Increase	Dave et al, NEJM, 2006
No	Up 1.5-5+ fold	84	Increase	Zhang et al, PNAS 2013
Yes	Up 4+ fold	73	Increase	Compagno et al, 2009, Nature

***Increase or decrease denotes the trend of fold change in MNK2 expression in comparison to normal tissue controls. The term 'split' observation represents a study where some samples exhibited an increase while others exhibited a decrease.**

MNK1

GCB/ABC classified?	Range of Expression	Sample Number	*Increase/decrease or split	Source
No	-8 to +3 fold	58	Split	Shipp et al, Nature Med, 2002
Yes	-5 to +0.5 fold	414	Decrease	Lenz et al, NEJM, 2008
No	About +1 fold	14	Increase	Klapper et al, Blood, 2008
No	-0.5 to +0.5 fold	11	Split	Brune et al, J Exp Med 2008
Yes	-4.5 to +1 fold	69	Decrease	Shaknovich et al, Blood 2010
No	-0.5 to +0.5 fold	23	Split	Lossos et al, PNAS, 2002

***Increase or decrease denotes the trend of fold change in MNK1 expression in comparison to normal tissue controls. The term 'split' observation represents a study where some samples exhibited an increase while others exhibited a decrease.**

Supplemental Table 2

eIF4E1 and eIF4E3 translatome

a: The list of significantly altered genes in eIF4E1 translatome

Symbol	(*Z*-ratio) E1TL-E1TR	(*Z*-ratio) E3TL-E3TR
A2LD1	1.63	1.33
ABHD11	1.68	0.89
ABHD12	1.63	0.36
ABL1	2.13	0.52
ACOT8	1.61	0.17
ADAM15	3.09	1.27
ADD1	1.62	0.87
ADORA2A	1.84	1.42
ADRBK1	2.07	1.41
ALG5	1.53	1.25
ALKBH5	1.73	0.95
ALKBH7	2.08	0.38
AMFR	1.75	0.15
ANKMY1	2.11	1.24
ANKRD16	1.60	1.05
ANXA11	1.52	1.13
AP3S2	1.82	1.32
APH1A	1.97	1.45
APH1A	1.57	0.88
ASB13	1.83	1.17
ASB7	2.05	1.42
ASCC2	1.58	1.33
ASNA1	1.54	1.00
ATF5	2.59	0.12
ATG9A	2.08	1.43
ATP5G3	1.59	1.15
ATP6V1G1	1.80	1.18
AXUD1	2.65	1.42
BAK1	1.57	1.26
BCL2L11	1.68	0.69
BCL2L11	1.61	0.70
BCL2L12	2.20	1.14
BLOC1S1	1.89	1.41
BOLA2	2.09	1.32
BRP44	1.66	1.19
C11orf17	2.14	0.89
C11orf31	1.76	1.71
C11orf73	2.12	1.29
C12orf66	1.65	0.90
C16orf13	1.72	1.07
C17orf101	1.79	1.36
C17orf101	1.55	1.35
C17orf101	1.53	1.18
C17orf101	1.82	1.38
C17orf95	1.54	0.61
C18orf56	1.84	0.33
C19orf28	2.09	1.12
C19orf47	1.69	1.49
C19orf60	1.76	1.19
C1orf159	1.78	1.23
C1orf43	2.04	-0.32
C1orf43	1.53	0.62
C1orf53	1.52	1.37
C1orf89	2.19	1.33
C20orf100	1.66	1.12
C21orf33	2.04	1.01
C21orf51	1.66	1.43
C21orf57	1.68	-0.48
C21orf70	2.47	0.53
C3orf54	1.70	0.90
C5orf32	2.33	1.12
C5orf39	2.63	1.11
C6orf148	1.89	1.46
C6orf89	1.85	1.29
C7orf11	2.32	1.26
C9orf23	1.67	1.08
C9orf37	2.12	0.48
C9orf69	2.08	1.33
C9orf89	2.25	0.29
C9orf9	1.80	-0.12
C9orf91	1.76	0.98
CARD17	1.89	0.57
CARKD	1.56	1.35
CCDC102A	2.38	1.40
CCDC106	2.30	1.18
CCDC130	1.65	1.36
CD163L1	1.65	1.24

CD99L2	2.08	1.25
CECR7	1.73	1.37
CENPM	3.28	2.74
CENPV	1.75	1.43
CENPV	2.46	1.42
CHCHD6	1.91	1.10
CHIC2	1.97	1.03
CHMP2A	1.61	-0.04
CINP	1.81	1.03
CLCN7	2.98	2.09
CMTM7	1.55	0.70
COQ9	1.76	0.97
CRADD	1.76	1.20
CRTC3	1.59	1.10
CSNK1D	2.00	1.12
CSNK1D	1.97	1.39
CSNK1G2	1.88	1.36
CUTA	1.86	1.42
CUTA	2.52	0.42
CXorf40A	2.41	1.76
CXorf40A	1.83	0.67
CXorf40B	2.18	1.49
CYHR1	1.68	0.81
DAP	1.57	1.31
DCTD	1.81	0.42
DCTD	1.72	1.48
DCTN5	1.65	1.04
DDA1	1.89	1.29
DDX12	2.07	2.13
DENND1A	1.94	-0.15
DENND1A	1.58	0.30
DEXI	3.01	1.58
DGCR6	1.69	1.27
DGCR6	1.76	1.17
DHDH	1.73	1.17
DLEU1	1.61	1.37
DNASE1L1	1.79	-0.05
DNPEP	1.67	1.39
DPH3	1.64	1.42
DPM3	1.55	0.80
DRAP1	1.63	1.39
DTD1	1.97	1.48
DTX2	1.96	1.31
DVL3	1.50	0.22

EBP	1.54	0.87
ECE2	3.08	1.96
ECGF1	1.53	0.04
ECSIT	1.50	1.30
EEF1B2	1.63	1.05
EEF1B2	1.68	1.31
EFNA4	2.24	0.77
EI24	1.55	0.04
EMD	1.63	0.83
EML3	2.09	1.49
ENDOG	1.60	0.73
ENSA	2.21	2.52
ENTPD6	2.26	0.39
ERGIC1	1.57	0.34
ESRRA	1.52	0.76
EVI5L	1.93	0.46
EXTL3	1.50	0.74
F8A2	1.89	1.06
FAHD2B	1.65	1.08
FAM116B	1.94	0.37
FAM125A	1.65	1.33
FAM128A	1.63	0.74
FAM134A	1.66	0.86
FAM14B	1.72	1.18
FAM160A2	1.66	1.36
FAM164C	1.73	1.46
FAM189B	1.52	0.96
FAM32A	1.71	1.45
FAM54B	1.52	0.36
FAM58A	1.82	1.31
FAM86A	1.51	0.70
FAM86B1	1.56	1.01
FANCA	2.06	1.30
FAT1	1.73	1.34
FBXO18	2.16	1.32
FBXW9	1.64	0.41
FGGY	1.54	0.90
FGGY	1.65	1.08
FLCN	1.93	1.41
FLJ10986	1.71	1.04
FLJ22222	1.51	0.89
FLJ38969	1.53	0.91
GBA2	1.54	0.42
GCHFR	1.73	-0.07

GLRX	1.91	1.41
GMFG	2.19	1.15
GMPPB	2.06	0.55
GNG5	1.61	1.07
GPBAR1	2.90	0.90
GPR114	2.67	-0.34
GPR137	1.93	0.65
GRB2	2.10	0.98
GSTZ1	2.20	0.98
H1FX	2.91	1.02
H2AFX	2.85	1.86
HAGH	1.56	0.64
HAGHL	1.54	-0.61
HAX1	1.54	0.13
HDDC2	1.81	1.41
HIGD2A	1.67	1.28
HINT3	1.67	0.93
HIST1H2AC	2.36	-0.16
HIST1H2BG	1.73	1.42
HIST1H2BJ	2.87	1.48
HIST1H2BK	2.69	1.18
HIST1H4J	2.15	1.50
HIST2H2AA3	2.39	-0.20
HIST2H3A	3.64	2.95
HIST2H3C	3.37	2.39
HIST3H2A	2.36	-0.03
HMG20B	2.43	1.28
HN1	2.73	1.26
HPCAL1	2.86	1.38
HSPC159	2.40	1.12
IFI27L2	1.70	1.07
IFI30	1.84	1.19
IL10RB	1.90	-0.07
IMMP1L	1.84	1.42
IQSEC3	1.67	0.81
IRAK1BP1	1.82	0.67
ISOC2	2.75	0.46
ITFG3	1.85	0.97
KCTD5	1.96	1.34
KDELR2	2.15	1.38
KIAA1310	1.52	1.15
KRT10	1.56	0.84

LAT	1.70	1.28
LCMT1	1.83	1.04
LLGL1	2.47	1.42
LOC100128689	2.07	1.08
LOC100128737	1.65	0.95
LOC100129201	1.92	1.18
LOC100129543	1.56	0.94
LOC100129890	1.52	1.42
LOC100129934	1.55	1.22
LOC100130009	2.61	1.70
LOC100130154	2.03	1.38
LOC100131866	1.95	1.16
LOC100132535	1.73	1.14
LOC100132564	2.27	-0.40
LOC100132728	1.50	1.71
LOC100132948	1.95	1.06
LOC100133517	1.61	1.05
LOC100134363	1.75	1.24
LOC136143	1.90	1.23
LOC148413	1.63	1.01
LOC201175	1.87	0.66
LOC205251	2.11	1.20
LOC253039	1.69	1.31
LOC255783	1.62	-0.16
LOC387791	1.52	0.90
LOC390298	1.60	0.71
LOC401238	1.75	1.32
LOC401847	1.51	1.23
LOC440525	1.81	1.47
LOC440957	1.60	1.29
LOC441408	1.64	0.36

LOC642393	1.82	1.17
LOC642502	2.90	1.20
LOC642934	1.72	-0.26
LOC643977	1.62	0.63
LOC644590	2.63	1.07
LOC644761	1.56	1.48
LOC644919	1.75	0.51
LOC645231	1.56	0.91
LOC645317	2.41	0.96
LOC645700	1.72	1.57
LOC645969	2.07	0.52
LOC647037	1.71	0.88
LOC648638	1.51	0.93
LOC648822	2.92	1.91
LOC649864	1.53	1.00
LOC650826	2.29	0.54
LOC652634	2.14	1.16
LOC652864	2.07	1.53
LOC653066	1.92	1.19
LOC653496	2.06	1.43
LOC654042	1.76	1.41
LOC654126	1.69	0.45
LOC728554	2.22	0.89
LOC728572	1.93	1.25
LOC728607	1.74	1.01
LOC728666	1.72	1.27
LOC728809	2.65	0.82
LOC728816	1.99	1.34
LOC728855	2.02	1.45
LOC728944	1.69	1.00

LOC729143	1.55	0.96
LOC729298	2.01	1.40
LOC729687	3.20	2.15
LOC729774	2.57	0.96
LOC729832	2.13	2.18
LOC729887	1.53	0.95
LOC730074	1.92	1.47
LOC730077	1.80	0.60
LOC730176	1.51	1.32
LOC730546	1.66	0.67
LONP1	1.55	1.12
LPCAT1	1.75	1.08
LRCH4	2.90	0.83
LRRC20	2.13	1.02
LY6E	2.74	1.44
LYPLA2	2.07	0.80
LYSMD4	1.68	1.05
MAD2L2	1.60	1.36
MAF1	1.71	0.79
MAP4K2	2.68	1.33
MAP6D1	1.76	1.35
MAPK3	2.20	0.97
MCOLN1	1.63	0.67
MED11	1.59	0.96
MED16	1.93	0.85
MESP1	1.61	1.23
MFN2	1.55	1.31
MGAT4B	1.57	0.95
MGC71993	1.62	1.27
MIF4GD	1.86	1.15
MKNK2	1.68	-0.33
MOBKL2C	1.54	0.39
MON1B	1.56	1.35
MRPL20	3.23	1.15
MRPL43	2.85	1.56
MRPS12	2.15	1.43
MRPS15	2.48	1.34
MSI2	1.73	0.77
MTP18	3.41	0.50
MYO19	1.76	1.40

MYST1	1.85	1.46
NACC1	1.62	1.48
NARF	1.97	1.15
NAT14	1.96	1.33
NCKIPSD	2.41	1.33
NDE1	1.83	0.88
NDST2	2.24	1.16
NHP2L1	1.93	0.74
NICN1	1.52	1.41
NKIRAS2	1.57	1.29
NLRX1	2.26	1.03
NME1	2.05	1.12
NME1-NME2	1.64	1.18
NME2	1.93	1.50
NOTCH2NL	1.57	0.30
NUDT18	2.40	0.47
OBFC1	1.67	0.58
OPA3	2.06	1.46
ORC6L	1.56	0.70
ORMDL3	1.58	1.07
OSCP1	1.92	1.37
PAGE1	1.51	-0.25
PAGE2B	1.62	1.08
PAOX	1.68	0.72
PARD6A	1.75	0.54
PARVB	2.25	1.27
PDE6D	1.67	0.77
PDF	2.35	1.44
PDXK	1.96	0.78
PEA15	1.59	1.09
PEX16	1.89	0.85
PEX7	2.05	0.75
PFKFB4	1.87	-1.48
PGAM4	2.05	0.95
PGAM4	2.07	1.07
PGAP3	1.79	1.10
PHF17	1.94	0.94
PIM3	1.68	1.09
PIN1	1.99	1.29
PLAGL1	1.52	0.76
PLAUR	1.54	1.29
PLAUR	1.59	1.38
PLD3	2.06	0.39
PLSCR3	1.51	0.94
PMM1	1.65	0.81
POLR2C	1.92	0.91
POLR2J	2.28	1.45
POMGNT1	1.70	1.14
PPCS	2.02	1.17
PPP1R12B	1.72	0.93
PRRG2	1.57	0.80
PRSS7	1.77	1.40
PSD4	2.32	1.17
PSENEN	1.74	1.04
PSMA7	1.73	0.76
PSMB8	1.59	1.36
PSMD13	1.80	1.11
PTRH1	2.59	1.43
PYCR1	1.58	1.06
RAB11FIP3	1.62	1.19
RAB1B	1.56	1.29
RAB33A	2.79	0.81
RAB40C	2.84	1.54
RAB5C	1.79	0.96
RAB5C	1.50	1.02
RBM38	2.35	1.19
RDH5	1.79	0.87
RER1	2.05	0.86
REXO1	1.64	1.60
RFC2	2.76	1.62
RHOC	1.58	0.54
RNF126	1.77	1.24
RNF167	2.82	1.55
RNF187	2.21	0.88
RNF41	1.76	0.72
RNF5	1.94	1.43
RNF5P1	2.02	1.45
RP11-529I10.4	1.96	1.00
RPESP	1.79	1.40
RPL41	1.60	1.44
RPRC1	1.58	0.90
RPS6KB2	2.38	1.10
S100A3	2.73	1.31
SCAMP3	1.64	1.44
SDHALP1	1.78	1.48
SFXN4	1.68	0.86

SFXN4	1.65	1.19
SH3GLB2	2.09	0.86
SIDT2	1.98	0.82
SIGMAR1	1.68	1.10
SIPA1	2.01	1.16
SLC10A3	1.63	1.31
SLC20A2	1.53	0.24
SLC25A14	1.51	0.48
SLC25A26	2.21	-0.10
SLC25A3	1.70	1.24
SLC25A42	2.06	0.94
SLC39A1	1.81	1.49
SLC39A4	2.23	1.19
SLC48A1	1.82	1.45
SLC7A14	2.51	1.47
SMPD1	1.66	0.75
SNAPC2	2.50	1.96
SNAPC5	1.51	0.68
SNRPN	2.45	0.79
SORT1	1.65	1.47
SPNS1	3.04	1.94
SPRYD3	1.90	1.10
SPSB3	1.69	0.82
SPTLC1	2.46	0.98
SRI	1.90	1.31
SRP14	1.86	1.30
SSR2	1.96	0.43
ST3GAL3	1.53	0.26
ST6GALNAC4	1.69	-0.03
ST6GALNAC4	1.91	0.85
ST8SIA1	2.19	0.69
ST8SIA1	1.93	0.86
STK19	1.93	0.51
STK19	1.60	0.39
STX11	1.56	1.33
STX8	2.02	1.46
SYTL3	2.04	0.64
TAPBP	1.87	0.67
TEAD4	2.51	0.34
TFAMP1	1.79	1.10
THOC3	1.54	1.43
TIMM23	2.50	0.50
TMC6	2.06	1.25

TMED9	2.16	1.01
TMEM128	1.62	0.63
TMEM141	2.98	1.89
TMEM149	1.96	1.33
TMEM185A	1.72	1.18
TMEM186	1.73	1.19
TMEM189	1.55	0.83
TMEM41A	2.28	0.60
TMEM55B	3.04	1.76
TMEM62	1.68	1.30
TMEM9	1.75	0.91
TMEM93	3.36	1.07
TMPRSS3	2.09	1.06
TMUB1	1.60	0.44
TNFRSF14	1.97	0.40
TNIP1	2.09	0.45
TOM1L2	1.61	1.18
TP53I13	2.41	1.34
TPI1	1.78	1.26
TPRG1L	2.45	1.30
TRAPPC6A	2.08	1.17
TRMT12	1.71	1.30
TRPT1	1.75	0.63
TSPAN3	1.74	1.14
TSPAN31	2.35	1.33
TSTD1	2.56	1.24
TYK2	1.65	1.41
UBE2D2	2.03	1.40
UBE2M	2.05	1.54
UNC119	1.67	0.41
UPLP	2.29	1.43
UPP1	2.12	1.48
UROS	1.59	1.01
USMG5	1.63	0.17
VEGFB	1.55	1.09
VEGFB	1.58	-0.06
WBP1	1.77	0.53
WBSCR16	2.12	0.45
WDR23	2.41	1.38
WDR54	2.05	1.12
WIBG	1.70	1.44
YIF1B	1.86	1.49
YIPF2	1.56	0.42

YIPF3	1.96	1.50
ZBTB45	1.92	1.79
ZDHHC8	2.21	1.10
ZDHHC9	1.66	0.77
ZFYVE21	1.53	0.87
ZMYM6	1.59	1.17
ZNF364	1.71	1.38
ZNF410	1.66	1.03
ZNF672	2.56	1.24
ZNF777	1.62	0.81
ZNF787	1.50	1.43
ZNF789	1.68	1.31
ZNF837	1.95	-0.07
ZNHIT3	1.54	0.68
ZSCAN5A	1.67	1.02

b. The list of significantly altered genes in eIF4E3 translatome

**Symbol**	**(*Z*-ratio) E1TL-E1TR**	**(*Z*-ratio) E3TL-E3TR**
ACOT7	1.42	1.94
ADCK1	1.49	1.82
ADPGK	0.91	1.71
ADRM1	1.35	2.84
ADRM1	1.15	2.39
AEN	1.28	1.83
ALG14	1.24	1.64
ALKBH2	1.08	1.72
ARL6IP4	1.44	1.57
ASF1B	0.90	2.03
ASRGL1	0.70	1.51
ATP5J	1.19	1.57
ATP5J	1.41	1.61
ATP5J	1.25	1.57
BANF1	0.93	1.64
BCS1L	1.43	1.65
BIRC5	1.25	1.51
BRI3BP	0.94	1.69
BSDC1	1.25	1.50
BUB3	0.61	1.64
C15orf24	1.31	1.55
C16orf57	1.20	1.98
C16orf75	1.07	1.82
C17orf53	1.22	1.91
C18orf45	1.40	1.70
C19orf40	1.45	1.62
C19orf43	1.13	1.66
C19orf52	1.03	1.67
C1orf52	1.35	1.60
C21orf70	2.15	1.71
C7orf40	1.23	2.36
C9orf140	1.12	1.91
CALM2	0.90	1.67
CAMK1G	0.78	1.82
CASP2	1.23	1.86
CASP9	1.20	1.56
CBR1	1.21	1.60
CCDC51	1.28	1.86
CCDC97	1.28	2.14
CCL3L3	1.16	1.93
CCNE1	1.22	1.70
CCRN4L	0.81	1.56
CDC20	0.65	1.54
CDC25A	0.90	2.19
CDC25B	1.44	2.30
CDC37	1.46	1.56
CDK2	0.29	1.70
CDK5R1	0.74	1.70
CDS2	1.31	1.75
CDT1	1.22	3.03
CEBPA	0.99	1.72
CENPN	1.06	1.68
CFL1	0.86	1.71
CHAF1A	0.57	1.72
CHPF2	1.28	1.76
CIAPIN1	1.22	1.67
CIDEB	1.11	1.54
CKLF	1.41	2.04
CKLF	1.49	2.06
CLDN14	1.36	1.57
CLN3	0.90	1.55
CMTM7	1.36	1.54

CNIH	0.76	1.61
CNIH	1.21	1.71
COMMD3	1.36	1.62
COX6C	1.20	1.52
CSF2RA	1.19	1.53
CTSB	1.41	1.62
CXorf64	1.38	2.37
CXXC1	1.27	2.24
DDOST	1.26	1.55
DDRGK1	1.26	1.71
DDX49	1.05	1.74
DDX51	1.01	1.92
DENND4B	1.09	1.57
DHRS2	0.91	2.21
DHRS2	1.06	2.19
DOLPP1	1.40	1.80
DOT1L	1.03	1.79
DPP9	1.39	1.51
DUS3L	1.35	1.99
DUSP2	0.79	1.53
EBNA1BP2	0.87	2.38
EEF1B2	1.26	1.81
EIF3I	0.94	2.14
ERAL1	1.10	1.58
ERCC1	1.18	1.55
EXOSC2	1.30	1.59
FABP5	1.03	2.66
FARS2	0.98	1.83
FAU	1.48	2.09
FBL	0.83	1.55
FBXO31	1.48	1.55
FEN1	-0.78	1.55
FHOD1	1.33	1.84
FKBPL	1.37	1.66
FLAD1	1.25	1.59
FLJ10661	1.15	1.97
FLJ20699	1.24	1.55
FTSJ1	1.38	1.77
GAGE12C	1.22	1.91
GAGE12E	1.28	1.75
GAGE12H	1.24	1.74
GAGE2B	1.10	2.16
GAGE6	1.07	2.01
GINS3	0.00	2.17

GMIP	1.41	1.58
GORASP1	1.33	1.59
GTF3C5	1.18	1.57
H3F3A	1.10	2.01
H3F3A	1.01	1.72
HAUS8	-0.01	1.52
HAUS8	0.70	2.17
HAUS8	0.46	1.54
HIST1H2AH	3.42	3.47
HIST1H2BE	2.30	1.73
HIST1H4C	1.44	2.70
HNRNPAB	1.36	2.40
HRASLS2	1.37	2.24
HSH2D	1.01	1.52
HSPE1	0.97	1.90
HTRA2	1.46	1.89
HYAL2	1.23	1.66
ID1	0.89	2.37
ID3	1.24	1.65
IGLL1	-0.03	2.47
IKBKE	1.18	2.07
IL6	-0.36	1.60
IQCC	1.41	1.61
IRAK1	1.50	1.97
ITGAE	1.26	1.81
ITM2C	0.64	2.09
ITM2C	0.54	1.92
KIAA0101	0.55	2.31
KSR1	1.55	2.08
KTI12	1.40	1.97
LDLRAP1	1.40	1.57
LMAN2	0.87	1.93
LOC100127993	1.13	1.80
LOC100128060	1.27	1.73
LOC100128060	1.38	1.70
LOC100129022	1.10	1.56
LOC100129552	1.38	1.69
LOC100130131	1.08	1.62
LOC100130224	1.34	1.63
LOC100130624	1.33	1.83
LOC100131196	1.37	2.11
LOC100132291	0.95	1.82
LOC387753	1.25	1.56
LOC388532	1.14	1.84

LOC391019	0.99	1.60
LOC402694	1.21	2.03
LOC440926	1.01	1.67
LOC642956	0.96	2.78
LOC643233	1.06	1.59
LOC643358	1.02	1.85
LOC644937	1.37	1.56
LOC645138	1.45	1.84
LOC646949	1.26	1.75
LOC648390	0.56	1.87
LOC648622	0.41	1.60
LOC648659	1.44	1.73
LOC648729	1.21	2.19
LOC651064	1.44	1.79
LOC651697	1.41	1.89
LOC653658	1.21	1.82
LOC653820	1.10	1.70
LOC653874	0.95	2.07
LOC728002	1.43	1.67
LOC728312	1.34	1.83
LOC728908	1.14	1.56
LOC729680	1.50	2.13
LOC729780	1.14	2.01
LOC729816	1.50	2.67
LOC730740	1.39	1.96
LOC731640	1.39	1.92
LRRC45	1.40	1.76
LRRC50	0.73	2.07
LRWD1	1.44	1.81
LSM3	1.28	1.60
LSM4	1.40	1.87
LSM5	1.42	1.62
Magmas	1.16	1.55
MCAT	1.23	1.54
MCM2	0.87	1.97
MDH2	1.26	1.76
METT11D1	0.55	1.79
MLLT6	1.10	1.58
MRI1	0.66	1.85
MRPL13	1.41	2.40
MRPL49	1.40	2.05
MRPL52	1.25	1.99
MTX1	1.46	1.95
NDUFB6	1.42	2.37

NFKBIA	1.36	1.73
NFKBIL2	0.45	1.56
NOL6	0.66	1.64
NPM3	1.37	2.16
NR2F2	1.32	1.75
NTHL1	1.48	2.19
NUBP1	1.24	2.02
NUBP2	1.39	1.63
NUDC	1.27	2.14
NUDCD3	1.14	1.57
ODC1	1.39	1.88
PARL	1.39	1.58
PARP12	1.09	2.61
PATE3	1.49	1.90
PCNA	-0.43	1.71
PEX11B	1.20	1.52
PFDN5	1.45	1.82
PFN1	1.46	1.73
PIGU	1.45	1.71
PKMYT1	1.27	1.94
PLD6	1.44	1.70
POLA2	0.92	2.09
POLR1C	0.95	2.15
POLR1C	0.43	2.05
POU2AF1	1.04	1.56
PPAN-P2RY11	0.99	2.16
PPP1R8	1.30	1.76
PPT2	1.30	1.94
PRCC	0.97	1.62
PRDX1	0.62	1.84
PRDX1	0.43	1.61
PRDX5	1.41	1.62
PRPF19	1.02	1.75
PRPF38A	1.05	1.53
PRR13	1.43	2.27
PRR3	1.42	1.64
PRR5	1.36	1.63
PSMA5	0.85	1.77
PSMB1	0.87	1.68
PSMB4	1.32	2.41
PSMB5	0.91	1.85
PSMB7	1.40	2.19
PSMB8	1.49	1.64
PSMD3	1.15	1.72

PSME1	1.09	1.53
PSME3	1.46	1.55
PSME3	1.21	1.53
PTTG1	1.06	1.73
PTTG3P	1.47	1.88
PUS1	1.16	1.62
PUSL1	1.29	1.97
PXMP2	1.28	1.64
RAD51L3	1.33	1.86
RAD54L	1.20	1.84
RAD9A	1.06	1.71
RAN	1.50	2.52
RASSF1	0.93	1.66
RASSF1	0.69	1.54
RBM42	0.80	1.91
RDH13	1.24	1.80
RECQL4	1.42	2.81
RNASEH2C	1.02	1.64
RNPS1	1.28	1.68
RPA2	0.76	1.65
RPL10A	0.93	1.62
RPP40	0.96	2.40
RPS15A	1.48	1.58
RPS26L	0.95	1.65
RPS26L	1.24	2.23
RPS27L	1.08	1.61
RPS27L	2.29	2.04
RPS6KA4	1.14	1.66
RPS7	0.76	1.52
RRP1	0.93	1.78
RSAD1	1.07	1.69
SAMD1	1.16	1.79
SAPS1	1.48	1.65
SCARNA10	1.43	2.31
SCLY	1.09	1.66
SEC61G	1.36	1.53
SFRS1	0.38	1.64
SFRS9	1.50	1.94
SFXN2	1.19	1.84
SH2D3A	1.46	1.68
SH3RF2	1.19	1.58
SIGMAR1	1.24	1.75
SIGMAR1	1.49	1.73
SLC25A4	1.23	1.51
SLC29A1	1.28	3.27
SLC29A1	1.17	2.86
SLC38A10	0.58	2.38
SLC38A5	0.58	1.56
SLC39A3	1.46	1.61
SNHG1	0.99	1.65
SNRPB	1.04	1.78
SNRPD2	1.13	1.99
SNRPF	1.41	2.53
SOD2	1.42	1.53
SPC25	0.79	1.55
SPCS1	1.40	1.92
SSBP1	1.12	1.84
ST3GAL1	0.65	1.58
STMN1	0.36	1.84
SUV39H1	1.44	2.32
SYK	1.39	1.64
TBCA	1.14	1.71
TH1L	1.31	1.70
TH1L	1.27	1.96
THOC4	1.36	1.57
THOC5	1.21	1.87
TM9SF1	1.01	1.55
TMSB4X	1.15	2.79
TNFRSF8	1.11	2.67
TOMM7	1.26	2.03
TOMM7	1.19	1.92
TPM3	1.67	1.59
TPST2	1.32	1.56
TRAF2	1.12	1.54
TRIM27	1.49	1.79
TRIM65	1.37	1.77
TRIP13	0.10	1.86
TRIP6	1.40	1.97
TRMT61A	0.85	1.93
TSFM	1.39	2.13
TTC38	1.35	1.75
TTC5	1.08	1.74
TUBA3C	0.35	2.39
TUBA3D	0.61	2.09
TUBG1	0.99	2.16
TXN	1.29	2.30
TXNDC14	1.33	1.94
TYMS	0.82	2.23

U2AF2	0.25	1.70
UBA52	1.36	1.76
UBXN1	1.43	1.78
UCHL5IP	0.55	1.53
UCHL5IP	0.58	1.50
UFD1L	1.14	1.57
UNG	0.73	2.27
VCX	-0.07	1.76
VCX-C	-0.25	1.95
VCX3A	0.17	1.81
VISA	1.37	1.59
VPS29	0.99	1.84
WDR77	1.38	2.31
WWOX	1.49	1.79
ZCCHC3	0.69	1.81
ZNF74	1.29	1.65
ZNF76	1.27	1.70

Supplemental Table 3

eIF4E1 candidate motif targets

#Ensembl.ID	Motif ID	Entrez Gene ID	Gene Name	Chromosome ID	Gene Set	Motif Position (s)
ENSG00000136240	1	11014	KDELR2	7	Target	56(-)
ENSG00000128626	1	6183	MRPS12	19	Target	154(+)
ENSG00000005194	1	57019	CIAPIN1	16	Bg	37(+)
ENSG00000088682	1	57017	COQ9	16	Target	47(-)
ENSG00000188580	1	154215	NKAIN2	6	Bg	16(-)
ENSG00000077092	1	5915	RARB	3	Bg	91(-)
ENSG00000156973	1	5147	PDE6D	2	Target	132(-)
ENSG00000125821	1	92675	DTD1	20	Target	25(-)
ENSG00000159461	1	267	AMFR	16	Target	132(-)
ENSG00000135452	1	6302	TSPAN31	12	Target	53(-)
ENSG00000147400	1	1069	CETN2	X	Bg	8(+)
ENSG00000166507	1	8509	NDST2	10	Target	93(+)
ENSG00000090674	1	57192	MCOLN1	19	Target	2(+)
ENSG00000099875	1	2872	MKNK2	19	Target	97(-)
ENSG00000137509	1	5547	PRCP	11	Bg	39(-)
ENSG00000115738	1	3398	ID2	2	Bg	79(-)
ENSG00000111371	1	81539	SLC38A1	12	Bg	152(-)
ENSG00000169019	1	54951	COMMD8	4	Bg	39(+)
ENSG00000110777	1	5450	POU2AF1	11	Bg	58(-)
ENSG00000205629	1	51451	LCMT1	16	Target	6(-)
ENSG00000184840	1	54732	TMED9	5	Target	9(-)
ENSG00000089693	1	8079	MLF2	12	Bg	29(+)
ENSG00000101182	1	5688	PSMA7	20	Target	12(+)
ENSG00000162885	1	148789	B3GALNT2	1	Bg	97(+)
ENSG00000101052	1	51098	IFT52	20	Bg	87(-)
ENSG00000176973	1	23625	FAM89B	11	Bg	98(-)
ENSG00000180198	1	751867	RCC1	1	Bg	156(-)
ENSG00000063241	1	79763	ISOC2	19	Target	21(-)
ENSG00000177374	1	3090	HIC1	17	Bg	48(+)
ENSG00000177302	1	7156	TOP3A	17	Bg	101(+)
ENSG00000145782	1	9140	ATG12	5	Bg	72(+)
ENSG00000240849	1	-	TMEM189	20	Target	14(-)
ENSG00000105223	1	23646	PLD3	19	Target	73(-)
ENSG00000092020	1	55012	PPP2R3C	14	Bg	13(-)
ENSG00000188785	1	147694	ZNF548	19	Bg	85(+)
ENSG00000166902	2	54948	MRPL16	11	Bg	52(+)
ENSG00000172315	2	112858	TP53RK	20	Bg	77(+)

ENSG00000146066	2	192286	HIGD2A	5	Target	40(-)
ENSG00000137944	2	56267	CCBL2	1	Bg	148(+)
ENSG00000197943	2	5336	PLCG2	16	Bg	85(+)
ENSG00000137880	2	2644	GCHFR	15	Target	85(-)
ENSG00000048162	2	51491	NOP16	5	Bg	181(+)
ENSG00000166783	2	9665	KIAA0430	16	Bg	63(+)
ENSG00000185885	2	8519	IFITM1	11	Bg	3(-)
ENSG00000101868	2	5422	POLA1	X	Bg	6(+)
ENSG00000169372	2	8738	CRADD	12	Target	42(+)
ENSG00000183628	2	8214	DGCR6	22	Target	89(+)
ENSG00000118655	2	64858	DCLRE1B	1	Bg	50(-)
ENSG00000007171	2	4843	NOS2	17	Bg	82(-)
ENSG00000186230	2	388567	ZNF749	19	Bg	78(+)
ENSG00000163923	2	116832	RPL39L	3	Bg	133(+)
ENSG00000133275	2	1455	CSNK1G2	19	Target	81(-)
ENSG00000174137	2	152877	FAM53A	4	Bg	189(-)
ENSG00000149577	2	51092	SIDT2	11	Target	21(+)
ENSG00000242485	2	-	MRPL20	1	Target	71(+)
ENSG00000149503	2	3619	INCENP	11	Bg	87(-)
ENSG00000139722	2	79720	VPS37B	12	Bg	180(-)
ENSG00000123374	2	1017	CDK2	12	Bg	111(-)
ENSG00000174021	2	2787	GNG5	1	Target	191(+)
ENSG00000164244	2	133619	PRRC1	5	Bg	105(-)
ENSG00000173581	2	29903	CCDC106	19	Target	21(+)
ENSG00000120645	2	731035	IQSEC3	12	Target	43(+)
ENSG00000165916	2	5702	PSMC3	11	Bg	42(+)
ENSG00000175662	2	146691	TOM1L2	17	Target	1(+)
ENSG00000144504	2	51281	ANKMY1	2	Target	8(+)
ENSG00000063601	2	8776	MTMR1	X	Bg	122(-)
ENSG00000197343	2	79027	ZNF655	7	Bg	72(+)
ENSG00000010671	2	695	BTK	X	Bg	122(+)
ENSG00000158555	2	81544	GDPD5	11	Bg	143(+)
ENSG00000148719	2	54788	DNAJB12	10	Bg	20(-)
ENSG00000198954	2	26128	KIAA1279	10	Bg	57(-)
ENSG00000107789	2	9562	MINPP1	10	Bg	32(-)
ENSG00000119725	2	57862	ZNF410	14	Target	2(+)
ENSG00000103342	2	2935	GSPT1	16	Bg	32(+)
ENSG00000134461	2	54522	ANKRD16	10	Target	137(-)
ENSG00000119632	2	83982	IFI27L2	14	Target	18(-)
ENSG00000103249	2	1186	CLCN7	16	Target	88(+)
ENSG00000198791	2	29883	CNOT7	8	Bg	13(+)

ENSG00000157823	2	10239	AP3S2	15	Target	163(-)
ENSG00000139168	2	85437	ZCRB1	12	Bg	16(+)
ENSG00000131652	2	79228	THOC6	16	Bg	48(+)
ENSG00000119574	2	84878	ZBTB45	19	Target	168(+)
ENSG00000088992	2	54997	TESC	12	Bg	82(-)
ENSG00000075218	2	51512	GTSE1	22	Bg	50(-)
ENSG00000105397	2	7297	TYK2	19	Target	171(+)
ENSG00000103145	2	54985	HCFC1R1	16	Bg	44(-)
ENSG00000198677	2	9652	TTC37	5	Bg	104(+)
ENSG00000143570	2	27173	SLC39A1	1	Target	24(-)
ENSG00000184481	2	4303	FOXO4	X	Bg	133(+)
ENSG00000100417	2	5372	PMM1	22	Target	51(+)
ENSG00000147872	2	123	PLIN2	9	Bg	16(+)
ENSG00000122140	2	51116	MRPS2	9	Bg	33(+)

Supplementary Table 4

eIF4E3 candidate motif targets

#Ensembl.ID	Motif ID	Entrez Gene ID	Gene Name	Chromosome ID	Gene Set	Motif Position (s)	
ENSG00000110107	1	27339	PRPF19	11	Target	175(-)	
ENSG00000117174	1	54680	ZNHIT6	1	Bg	26(+)	
ENSG00000138495	1	10063	COX17	3	Bg	79(-)	
ENSG00000005156	1	3980	LIG3	17	Bg	45(+)	
ENSG00000071894	1	29894	CPSF1	8	Bg	68(+)	
ENSG00000188612	1	-	SUMO2	17	Bg	60(-)	
ENSG00000100206	1	11144	DMC1	22	Bg	96(-)	
ENSG00000228532	1	-	RP1-241P17.4	X	Bg	58(-)	
ENSG00000108953	1	7531	YWHAE	17	Bg	90(+)	
ENSG00000114346	1	1894	ECT2	3	Bg	40(+)	
ENSG00000161914	1	115950	ZNF653	19	Bg	20(-)	
ENSG00000196141	1	26010	SPATS2L	2	Bg	58(-)	
ENSG00000188542	1	285193	DUSP28	2	Bg	9(+)	
ENSG00000125885	1	84515	MCM8	20	Bg	27(+)	
ENSG00000101856	1	10857	PGRMC1	X	Bg	28(+)	
ENSG00000249709	1	-	ZNF564	19	Bg	107(-)	
ENSG00000148019	1	84131	CEP78	9	Bg	51(+)	
ENSG00000160051	1	55721	IQCC	1	Target	10(-)	
ENSG00000078674	1	5108	PCM1	8	Bg	181(-)	
ENSG00000120832	1	80298	MTERFD3	12	Bg	31(-)	
ENSG00000198176	1	7027	TFDP1	13	Bg	8(+)	
ENSG00000099901	1	5902	RANBP1	22	Bg	129(+)	
ENSG00000198146	1	54989	ZNF770	15	Bg	84(+)	
ENSG00000186153	1	51741	WWOX	16	Target	21(-)	
ENSG00000018510	1	8540	AGPS	2	Bg	6(+)	
ENSG00000014138	1	23649	POLA2	11	Target	175(-)	
ENSG00000140374	1	2108	ETFA	15	Bg	22(+)	
ENSG00000157184	1	1376	CPT2	1	Bg	189(+)	
ENSG00000130517	1	54858	PGPEP1	19	Bg	27(+)	
ENSG00000135392	1	85406	DNAJC14	12	Bg	80(+)	
ENSG00000071462	1	114049	WBSCR22	7	Bg	25(+)	
ENSG00000168646	1	8313	AXIN2	17	Bg	7(-)	
ENSG00000123349	1	5204	PFDN5	12	Target	175(-)	
ENSG00000113569	1	9631	NUP155	5	Bg	95(-)	
ENSG00000088247	1	8570	KHSRP	19	Bg	16(-)	
ENSG00000130489	1	9997	SCO2	22	Bg	32(-)	

ENSG00000088205	1	8886	DDX18	2	Bg	93(-)	
ENSG00000076248	1	7374	UNG	12	Target	19(+)	
ENSG00000173530	1	8793	TNFRSF10D	8	Bg	61(-)	
ENSG00000169021	1	7386	UQCRFS1	19	Bg	92(-)	
ENSG00000003756	1	10181	RBM5	3	Bg	141(-)	
ENSG00000139684	1	2098	ESD	13	Bg	139(-)	
ENSG00000213445	1	6494	SIPA1	11	Bg	118(+)	
ENSG00000125445	1	51081	MRPS7	17	Bg	65(-)	
ENSG00000127616	1	6597	SMARCA4	19	Bg	54(-)	
ENSG00000197451	1	3182	HNRNPAB	5	Target	107(-)	
ENSG00000156502	1	6832	SUPV3L1	10	Bg	31(+)	
ENSG00000122779	1	8805	TRIM24	7	Bg	23(+)	
ENSG00000141873	1	29985	SLC39A3	19	Target	101(+)	
ENSG00000119986	1	60370	AVPI1	10	Bg	31(-)	
ENSG00000135164	1	9988	DMTF1	7	Bg	143(-)	
ENSG00000127564	1	9088	PKMYT1	16	Target	72(-)	
ENSG00000117751	1	5511	PPP1R8	1	Target	32(+)	
ENSG00000117748	1	6118	RPA2	1	Target	42(-)	
ENSG00000115539	1	79031	PDCL3	2	Bg	161(-)	
ENSG00000166226	1	10576	CCT2	12	Bg	32(+)	
ENSG00000152076	1	91409	CCDC74B	2	Bg	19(+)	
ENSG00000103502	1	10423	CDIPT	16	Bg	42(+)	
ENSG00000136810	1	7295	TXN	9	Target	141(-)	
ENSG00000112893	1	4124	MAN2A1	5	Bg	109(-)	
ENSG00000163516	1	55139	ANKZF1	2	Bg	139(-)	
ENSG00000168374	1	378	ARF4	3	Bg	60(+)	
ENSG00000166169	1	27343	POLL	10	Bg	14(+)	
ENSG00000166166	1	115708	TRMT61A	14	Target	47(-)	
ENSG00000117632	1	3925	STMN1	1	Target	8(-)	
ENSG00000132305	1	10989	IMMT	2	Bg	163(+)	
ENSG00000124702	1	116138	KLHDC3	6	Bg	63(-)	
ENSG00000173209	1	130872	AHSA2	2	Bg	79(-)	
ENSG00000085274	1	55892	MYNN	3	Bg	62(+)	
ENSG00000139343	1	6636	SNRPF	12	Target	26(-)	
ENSG00000115317	1	27429	HTRA2	2	Target	71(-)	
ENSG00000173171	1	4580	MTX1	1	Target	79(+)	
ENSG00000196683	1	54543	TOMM7	7	Target	54(+)	
ENSG00000099341	1	5714	PSMD8	19	Bg	55(+)	
ENSG00000180263	1	55785	FGD6	12	Bg	74(-)	
ENSG00000126768	1	10245	TIMM17B	X	Bg	30(+)	
ENSG00000120137	1	79646	PANK3	5	Bg	46(-)	

ENSG00000180198	1	751867	RCC1	1	Bg	14(+)	
ENSG00000077420	1	54518	APBB1IP	10	Bg	70(+)	
ENSG00000167565	1	29946	SERTAD3	19	Bg	71(+)	
ENSG00000169714	1	7555	CNBP	3	Bg	38(-)	
ENSG00000198677	1	9652	TTC37	5	Bg	66(+)	
ENSG00000176890	1	7298	TYMS	18	Target	45(+)	73(+)
ENSG00000109689	1	57620	STIM2	4	Bg	137(-)	
ENSG00000109618	1	51091	SEPSECS	4	Bg	25(-)	
ENSG00000181704	1	286451	YIPF6	X	Bg	64(+)	
ENSG00000178980	1	6415	SEPW1	19	Bg	64(-)	
ENSG00000003056	1	4074	M6PR	12	Bg	127(-)	
ENSG00000077254	2	23032	USP33	1	Bg	54(-)	
ENSG00000172339	2	199857	ALG14	1	Target	6(+)	
ENSG00000216649	2	729431	GAGE12E	X	Target	15(+)	
ENSG00000164713	2	25798	BRI3	7	Bg	22(-)	
ENSG00000053254	2	1112	FOXN3	14	Bg	46(-)	
ENSG00000177192	2	80324	PUS1	12	Target	104(+)	
ENSG00000105135	2	10994	ILVBL	19	Bg	41(-)	
ENSG00000198483	2	148741	ANKRD35	1	Bg	20(+)	
ENSG00000213930	2	2592	GALT	9	Bg	25(-)	
ENSG00000077152	2	29089	UBE2T	1	Bg	125(+)	
ENSG00000162482	2	22977	AKR7A3	1	Bg	77(-)	
ENSG00000125910	2	8698	S1PR4	19	Bg	7(+)	
ENSG00000164611	2	9232	PTTG1	5	Target	3(+)	
ENSG00000101945	2	6839	SUV39H1	X	Target	63(+)	
ENSG00000189064	2	729447	GAGE2A	X	Bg	26(+)	
ENSG00000173930	2	353189	SLCO4C1	5	Bg	17(+)	
ENSG00000165025	2	6850	SYK	9	Target	64(+)	
ENSG00000009413	2	5980	REV3L	6	Bg	179(+)	
ENSG00000112149	2	9308	CD83	6	Bg	59(-)	
ENSG00000114315	2	3280	HES1	3	Bg	37(-)	
ENSG00000125898	2	83541	FAM110A	20	Bg	59(-)	
ENSG00000077092	2	5915	RARB	3	Bg	177(-)	
ENSG00000100162	2	79019	CENPM	22	Bg	69(+)	
ENSG00000114268	2	5210	PFKFB4	3	Bg	47(+)	
ENSG00000143179	2	7371	UCK2	1	Bg	91(-)	
ENSG00000125743	2	6633	SNRPD2	19	Target	74(+)	
ENSG00000012061	2	2067	ERCC1	19	Target	110(+)	
ENSG00000237671	2	-	GAGE12C	X	Target	25(+)	
ENSG00000086200	2	51194	IPO11	5	Bg	92(-)	154(-)
ENSG00000054611	2	25771	TBC1D22A	22	Bg	76(-)	

ENSG00000104331	2	54928	IMPAD1	8	Bg	8(-)	
ENSG00000145220	2	55646	LYAR	4	Bg	6(+)	
ENSG00000067064	2	3422	IDI1	10	Bg	110(-)	
ENSG00000154582	2	6921	TCEB1	8	Bg	72(-)	
ENSG00000014138	2	23649	POLA2	11	Target	90(+)	
ENSG00000144747	2	7110	TMF1	3	Bg	109(-)	
ENSG00000080802	2	4850	CNOT4	7	Bg	21(-)	
ENSG00000149547	2	9538	EI24	11	Bg	139(+)	
ENSG00000135372	2	55226	NAT10	11	Bg	168(-)	
ENSG00000198001	2	51135	IRAK4	12	Bg	17(+)	
ENSG00000105976	2	4233	MET	7	Bg	123(+)	
ENSG00000101596	2	23347	SMCHD1	18	Bg	12(+)	
ENSG00000101558	2	9218	VAPA	18	Bg	32(+)	
ENSG00000204152	2	653252	TIMM23B	10	Bg	72(-)	100(-)
ENSG00000175756	2	54998	AURKAIP1	1	Bg	14(+)	
ENSG00000183336	2	654483	BOLA2	16	Bg	121(-)	
ENSG00000258728	2	-	RP11-195F19.29	9	Bg	43(-)	
ENSG00000108506	2	57508	INTS2	17	Bg	160(+)	
ENSG00000028116	2	7444	VRK2	2	Bg	160(+)	
ENSG00000224902	2	-	GAGE12H	X	Target	25(+)	
ENSG00000197451	2	3182	HNRNPAB	5	Target	66(+)	
ENSG00000156508	2	1915	EEF1A1	6	Bg	30(+)	
ENSG00000205777	2	2578	GAGE1	X	Bg	54(+)	
ENSG00000141873	2	29985	SLC39A3	19	Target	24(-)	
ENSG00000100926	2	10548	TM9SF1	14	Target	56(+)	
ENSG00000168495	2	661	POLR3D	8	Bg	55(+)	
ENSG00000127564	2	9088	PKMYT1	16	Target	174(-)	
ENSG00000160999	2	-	SH2B2	7	Bg	92(-)	
ENSG00000176024	2	79898	ZNF613	19	Bg	84(+)	
ENSG00000164045	2	993	CDC25A	3	Target	185(-)	
ENSG00000134690	2	55143	CDCA8	1	Bg	183(+)	
ENSG00000227488	2	-	GAGE12D	X	Bg	25(+)	
ENSG00000167965	2	64223	MLST8	16	Bg	140(-)	
ENSG00000132432	2	23480	SEC61G	7	Target	156(+)	
ENSG00000034677	2	25897	RNF19A	8	Bg	15(-)	
ENSG00000127452	2	54850	FBXL12	19	Bg	79(+)	
ENSG00000105671	2	54555	DDX49	19	Target	22(-)	
ENSG00000113240	2	57396	CLK4	5	Bg	49(+)	
ENSG00000025293	2	51230	PHF20	20	Bg	14(+)	
ENSG00000068305	2	4205	MEF2A	15	Bg	96(-)	
ENSG00000198925	2	79065	ATG9A	2	Bg	158(-)	

ENSG00000120314	2	54853	WDR55	5	Bg	182(+)	
ENSG00000205542	2	7114	TMSB4X	X	Target	27(+)	
ENSG00000117592	2	9588	PRDX6	1	Bg	82(-)	
ENSG00000224659	2	-	GAGE12J	X	Bg	39(+)	
ENSG00000172731	2	55222	LRRC20	10	Bg	71(-)	
ENSG00000030110	2	578	BAK1	6	Bg	187(-)	
ENSG00000175387	2	4087	SMAD2	18	Bg	155(-)	
ENSG00000112658	2	6722	SRF	6	Bg	4(+)	
ENSG00000215269	2	645051	GAGE12G	X	Bg	25(+)	
ENSG00000198839	2	11179	ZNF277	7	Bg	42(+)	
ENSG00000157911	2	5192	PEX10	1	Bg	8(-)	
ENSG00000131747	2	-	TOP2A	17	Bg	100(+)	
ENSG00000168172	2	84376	HOOK3	8	Bg	133(-)	
ENSG00000131725	2	54521	WDR44	X	Bg	164(-)	
ENSG00000162851	2	64216	TFB2M	1	Bg	125(+)	
ENSG00000057935	2	57504	MTA3	2	Bg	12(-)	
ENSG00000187231	2	91404	SESTD1	2	Bg	60(-)	
ENSG00000197008	2	7697	ZNF138	7	Bg	161(-)	
ENSG00000124562	2	6631	SNRPC	6	Bg	54(+)	
ENSG00000102796	2	79758	DHRS12	13	Bg	69(+)	
ENSG00000163257	2	54876	DCAF16	4	Bg	181(+)	
ENSG00000001084	2	2729	GCLC	6	Bg	157(+)	
ENSG00000112511	2	5252	PHF1	6	Bg	134(-)	
ENSG00000184557	2	9021	SOCS3	17	Bg	127(+)	
ENSG00000151233	2	283464	GXYLT1	12	Bg	30(+)	
ENSG00000115137	2	51277	DNAJC27	2	Bg	165(+)	
ENSG00000007541	2	9091	PIGQ	16	Bg	141(-)	
ENSG00000145723	2	54826	GIN1	5	Bg	9(+)	
ENSG00000236362	2	-	GAGE12F	X	Bg	25(+)	
ENSG00000138594	2	29766	TMOD3	15	Bg	31(-)	
ENSG00000189241	2	7259	TSPYL1	6	Bg	51(+)	
ENSG00000169683	2	648576	LRRC45	17	Target	115(+)	

Supplementary Table 5

Nucleotide frequency table indicating the rate of nucleotide occurrence in each position of a motif

	Motif 2	0	1	2	3	4	5	6	7	8	9
	A	0.7092	0.0628	0.0000	0.0626	0.1846	0.0000	0.0000	0.9387	0.0959	0.0000
	C	0.1911	0.0000	0.0000	0.8708	0.0000	0.0000	0.0000	0.0000	0.6501	0.0733
	G	0.0997	0.6437	0.0000	0.0667	0.7515	1.0000	1.0000	0.0000	0.0000	0.6139
	T	0.0000	0.2935	1.0000	0.0000	0.0639	0.0000	0.0000	0.0613	0.2541	0.3128
**eIF4E1**											
	**Motif 1**	**0**	**1**	**2**	**3**	**4**	**5**	**6**	**7**	**8**	**9**
	A	0.9279	0.0000	0.0000	0.0000	0.0000	0.3342	0.0000	0.0000	0.0000	0.9279
	C	0.0000	1.0000	0.0000	0.0892	0.6077	0.0000	0.9206	0.9206	0.6803	0.0000
	G	0.0000	0.0000	1.0000	0.1537	0.1515	0.6117	0.0794	0.0794	0.3197	0.0000
	T	0.0721	0.0000	0.0000	0.7571	0.2408	0.0541	0.0000	0.0000	0.0000	0.0721

	Motif 2	0	1	2	3	4	5	6	7	8	9
	A	0.0000	0.0000	0.0707	0.0000	0.0000	0.2224	0.2399	0.0000	0.0000	0.1032
	C	0.7467	0.0000	0.0000	0.1253	0.0000	0.0000	0.0838	0.1407	0.3831	0.8968
	G	0.0000	1.0000	0.6188	0.6653	0.3225	0.7776	0.6764	0.0000	0.0000	0.0000
	T	0.2533	0.0000	0.3105	0.2094	0.6775	0.0000	0.0000	0.8593	0.6169	0.0000
**eIF4E3**											
	**Motif 1**	**0**	**1**	**2**	**3**	**4**	**5**	**6**	**7**	**8**	**9**
	A	0.0000	0.0000	0.0862	0.0000	0.0000	1.0000	0.8088	0.6253	0.1106	0.3491
	C	0.0840	1.0000	0.0000	1.0000	1.0000	0.0000	0.0956	0.0739	0.4641	0.0000
	G	0.9160	0.0000	0.6083	0.0000	0.0000	0.0000	0.0956	0.0000	0.0000	0.6509
	T	0.0000	0.0000	0.3055	0.0000	0.0000	0.0000	0.0000	0.3008	0.4253	0.0000

Supplementary Table 6

Comparison of eIF4E1 and eIF4E3-driven 5'UTR motifs to TOP motif

a. Nucleotide frequency table of the TOP motif (Thoreen et al, 2012 and Eliseeva et al 2013).

Motif	0	1	2	3	4	5	6	7	8	9	10	11	12	13
A	0.0561	0.0000	0.0701	0.0047	0.0047	0.0000	0.0187	0.0421	0.0467	0.0000	0.0047	0.0000	0.0280	0.1075
C	0.3645	0.6916	0.0467	0.9393	0.2150	0.1869	0.4813	0.8411	0.4299	0.2710	0.1963	0.3738	0.6495	0.4112
G	0.3925	0.0000	0.1682	0.0374	0.0327	0.1075	0.0514	0.0140	0.0047	0.0841	0.0374	0.0514	0.0561	0.3785
T	0.1869	0.3084	0.7150	0.0187	0.7477	0.7056	0.4486	0.1028	0.5187	0.6449	0.7617	0.5748	0.2664	0.1028

b. Divergence scores for each motif (with and without reverse complementing) when compared to the TOP motif

Raw divergence value	Motif	Direction	TOP-like motif sub-sequences				
			1..10	2..11	3..12	4..13	5..14
eIF4E1	Motif 2	same	0.7717	0.9712	0.9413	0.8693	0.7667
		opposite	0.6789	0.8394	0.7773	0.6715	0.7471
	Motif 1	same	0.7824	0.8270	0.7559	0.9096	0.8890
		opposite	0.8226	0.8844	0.8208	0.9845	0.8686
eIF4E3	Motif 2	same	0.7798	0.7108	0.7697	0.6566	0.7651
		opposite	0.7501	0.8529	0.8634	0.8222	0.7383
	Motif 1	same	0.7122	1.0005	0.8072	0.8054	0.7873
		opposite	0.8005	0.7682	0.8371	0.8220	0.8490

Minimum divergence value	Motif	TOP motif Divergence Score	Direction	TOP motif sub-sequence
eIF4E1	Motif 2	0.6715	opposite	4 .. 13
	Motif 1	0.7559	same	3 .. 12
eIF4E3	Motif 2	0.6566	same	4 .. 13
	Motif 1	0.7122	same	1 .. 10

Supplementary Table 7

a) shRNA sequences

**shRNA target (catalog number)**	**Sequences**
MNK1 (TRCN0000314869)	CCGGTGCTCCAGTCACACCTTATAGCTCGA GCTATAAGGTGTGACTGGAGC
MNK2 (TRCN0000006098)	CCGGCCTAGAGCTGATTGAGTTCTTCTCGA GAAGAACTCAATCAGCTCTAGGTTTTT
MNK2 (TRCN0000342226)	CCGGCATGTGTTAATGTTACGATGTCTCGA GACATCGTAACATTAACACATGTTTTTG
eIF4E1 (TRCN0000310424)	CCGGCGGCTGATCTCCAAGTTTGATCTCGA GATCAAACTTGGAGATCAGCCGTTTTTG
eIF4E3 (TRCN0000148696)	CCGGCCATGAAGAGCATCATGCTTTCTCGA GAAAGCATGATGCTCTTCATGGTTTTTTG
eIF4E3 (TRCN0000146957)	CCGGCCAATAGGAAACCTGATGTTTCTCGA GAAACATCAGGTTTCCTATTGGTTTTTTG
eIF4E3 (TRCN0000147940)	CCGGGCAGCAGATGATGAAGTAATACTCG AGTATTACTTCATCATCTGCTGCTTTTTTG
eIF4E3 (TRCN0000147703)	CCGGGTTTGGAAAGAGTTGCTGTTACTCGA GTAACAGCAACTCTTTCCAAACTTTTTTG

b) Primer sequences used in RT-qPCR analyses

**Target Gene**	**Primers**
GAPDH	*For- CGGAGTCAACGGATTTGGTCGTA, Rev-AGCCTTCTCCATGGTGGTGAAGAC*
MNK1	*For-TGCTTTTGCTTCTGGATGT Rev-AGAAACAAGCAGGGCACAGT*
MNK2	*For-AGTCCCCGTTGAGTTTGAT Rev-AAAGCCGGAAAACATCCTCT*
eIF4E1	*For-AGTGACCTCGATCGCTTTTG Rev- ACATTAACAACAGCGCCACA*
DTD1	*For-TGAGGATGAGAGTGGGAAGC Rev-GCTCCGGCCTGTATGTTTTA*
DGCR6	*For- GCTGCCTTTCTGGTCACACT Rev- CAGCAGAGCACACTGGAGAA*
FLCN	*For- CCAGCTCTTCAGCATTGTCC Rev- GAGGTAGATCCGGTCCATCA*
Bub3	*For-AATGCTGGGACCTTCTCTCA Rev-GGCTTGGGTCCAAATACTCA*
LSM4	*For- GATGCCCGAGTGCTACATC Rev- CTGTCTGCCAGGCTTCTTCT*
FAU	*For- CCCAGGAGCTACACACCTTC Rev- TTGGCCACCTTAGGAGTCTG*
